# The AKAP12-PKA axis regulates lipid homeostasis during alcohol-associated liver disease

**DOI:** 10.1038/s41392-025-02202-1

**Published:** 2025-04-09

**Authors:** Chandana Thimme Gowda, Mallikarjuna Siraganahalli Eshwaraiah, Jiaohong Wang, Youngyi Lim, Maria Lauda Tomasi, Nirmala Mavila, Komal Ramani

**Affiliations:** 1 Karsh Division of Gastroenterology and Hepatology, Cedars-Sinai Medical Center, Los Angeles, CA USA; 2 Applied Cell Biology Division, Department of Biomedical Sciences, Cedars-Sinai Medical Center, Los Angeles, CA USA

**Keywords:** Molecular biology, Cell biology

## Abstract

Disrupted lipogenic signaling and steatosis are key features of alcohol-associated liver disease (ALD). A-kinase anchoring protein 12 (AKAP12) is a scaffolding partner of the cAMP-dependent protein kinase, PKA that controls its spatiotemporal localization. Activation of PKA by cAMP inhibits lipogenesis and facilitates fatty acid oxidation (FAO). The goal of this work is to examine how AKAP12’s PKA-anchoring ability regulates outcomes of alcohol-associated steatosis. Crosslinking proteomics identified PKA and its lipogenic substrates as interacting partners of AKAP12. Alcohol exposure diminished AKAP12’s interaction with PKA regulatory subunits and PKA substrates, acetyl CoA carboxylase (ACC1), pyruvate dehydrogenase (PDHA) and adipose triglyceride lipase (ATGL). Alcohol inhibited PKA activity and increased triglyceride content in human hepatocytes. Forced expression of *AKAP12* restored alcohol suppressed PKA activation and inhibited lipid accumulation, whereas silencing had the reverse effect. Since *AKAP12* sustained PKA activity, we evaluated whether the AKAP12-PKA scaffold was important in lipid homeostasis. Inhibition of AKAP12-PKA interaction by CRISPR deletion of AKAP12’s PKA binding domain in cultured hepatocytes or in mouse models of ALD dramatically suppressed PKA activity, enhanced ACC1 activity demonstrated by reduced inhibitory phosphorylation, increased lipid accumulation and reduced FAO in hepatocytes. Overexpression of *AKAP12* in mouse livers sustained PKA activation, diminished basal and alcohol potentiated triglyceride content, and regulated inflammatory signaling altered by alcohol. Mechanistically, we discovered that alcohol enhanced the inhibitory activity of a kinase, serine/threonine-protein kinase 25 (STK25) on PKA that regulated its interaction with AKAP12. In conclusion, the AKAP12-PKA scaffold controls lipogenic signaling, disruption of which favors steatosis during ALD.

## Introduction

Steatosis or fatty liver is one of the early changes in response to moderate to high alcohol drinking.^1^ Individuals with alcohol-dependent fatty liver are susceptible to advanced liver pathologies, including alcohol-associated steatohepatitis (ASH), liver fibrosis, and liver cirrhosis.^2,3^ Therefore, hepatic steatosis is an essential factor in contributing to advanced alcohol-associated liver disease (ALD). Disruption of lipid homeostasis could be at the level of fatty acid biosynthesis, uptake, oxidation, export, and lipolysis, all of which can contribute to liver fat.^4^

Protein kinase A (PKA) is heterotetrameric enzyme consisting of two regulatory subunits (RI/RII-α or RI/RII-β) and two catalytic subunits (isotypes-Cα, Cβ, Cγ, PRKX).^5^ The release of catalytic subunits from this heterotetramer induces PKA catalytic activity in a cyclic-AMP (cAMP)-dependent manner.^5^ PKA/cAMP signaling plays an important role in lipid homeostasis in the liver.^6^ Increased cAMP levels suppress lipogenesis through PKA-dependent inhibitory phosphorylation of fatty acid biosynthesis enzymes, acetyl CoA carboxylase (ACC1) and pyruvate dehydrogenase (PDHA).^6,7^ The PKA target, ACC1 is the enzyme involved in the first step of fatty acid biosynthesis.^8^ It catalyzes the carboxylation of acetyl-CoA to produce malonyl-CoA, a key metabolite facilitating de novo lipogenesis.^9^ PKA and AMPK are known to phosphorylate ACC1^7,10^ and PKA was previously demonstrated to activate the catalytic subunit of AMPK (PRKAA1 or acetyl CoA carboxylase kinase) which promoted inhibitory phosphorylation of ACC1.^11^ Blocking ACC1 inhibitory phosphorylation was shown to enhance de novo lipogenesis in MASLD and promote hepatocellular carcinoma (HCC) lesions.^9^ Inhibiting ACC1 activity can suppress hepatic steatosis.^12^ The cAMP responsive transcription factor, CREB is positively regulated through ser-133 PKA-dependent phosphorylation which facilitates its nuclear translocation and DNA-binding activity.^13^ PKA-activated CREB inhibits hepatic expression of lipid synthesis promoting genes, peroxisome–proliferator activator receptor gamma (PPAR-γ)^14^ and sterol regulatory element-binding protein-1c (SREBP-1C).^15^ CREB-mediated activation of peroxisome proliferator-activated receptor-γ (PPARγ) coactivator-1α (PGC-1 α) along with CREB-dependent PPAR-γ suppression can enhance mitochondrial fatty acid oxidation (FAO).^16^ Phospho-activation of CREBH at ser-133 suppresses plasma triglyceride levels through transcriptional activation of genes involved in triglyceride metabolism.^17^ PKA-dependent CREB activation is also involved in inhibiting inflammatory signaling through NF-κB.^18^ Inhibition of hepatic PKA catalytic subunit activity in transgenic mice overexpressing a pseudopeptide inhibitor, PKI-α aggravates lipid accumulation under conditions of high-fat diet feeding in mice.^19^ Alcohol exposure dysregulates PKA signaling, decreases cAMP levels and suppresses CREB activation.^20,21^ Overall these studies support an essential function of PKA in regulating lipid metabolism.

AKAP12 is a scaffolding protein that belongs to the A-kinase anchoring protein family. AKAP12 contains an amphipathic alpha-helical PKA-binding domain that interacts with the N-terminal dimerization and docking domain of PKA regulatory subunits.^22^ Functional roles of AKAP12’s PKA scaffolding activity are established in cell-based systems. AKAP12 facilitates PKA localization at sites of the β-2 adrenergic receptor (ADRB2) on the plasma membrane causing PKA-dependent ADRB2 de-sensitization.^23^ The presence of the PKA-AKAP12 scaffold at the ADRB2 site allows AKAP12 to communicate with ADRB2 and influence receptor re-sensitization/plasma membrane recycling.^24–26^ In addition to PKA, AKAP12 binds to other signaling proteins, such as cAMP phosphodiesterases, protein kinase C (PKC), SRC kinase, phosphatases, and cytoskeletal proteins.^26^ As a result of its large signalosome, AKAP12 can coordinate multiple functions, such as control of cell motility, cell morphology, cytoskeletal reorganization, regulation of cell cycle progression, and inhibition of SRC-mediated oncogenic signaling.^27^ In the liver, cell-specific interactions of AKAP12 are observed. We recently published unique interactions of AKAP12 with the collagen chaperone, heat shock protein 47 (HSP47) in hepatic stellate cells (HSCs) that drives outcomes of liver fibrosis.^28^ Other investigators have demonstrated that AKAP12-dependent regulation of portal fibroblasts and liver sinusoidal endothelial cells plays an important role in liver fibrosis resolution.^29^ We published previously that AKAP12’s interactions with PKC and HSP47 are suppressed by alcohol exposure in HSCs.^30^ The overall expression of AKAP12 is known to be influenced during liver disease. AKAP12 expression is decreased during ALD, liver fibrosis, cirrhosis, and HCC.^28–31^ Recently, studies of hepatocyte-specific *AKAP12-*knock out mice revealed that AKAP12 plays an important role in controlling inflammation during acute liver injury through its modulation of the PI3-kinase/AKT/ Proprotein convertase subtilisin/kexin type 6 (PCSK6) pathway.^32^

Although PKA has a long-standing role in controlling lipid metabolism and is known to be regulated by AKAP scaffolding,^22^ the potential role of AKAP12 as a modulator of PKA-dependent lipogenic signaling in the liver has not been assessed. The purpose of this work is to investigate how AKAP12 scaffolding affects PKA’s ability to control lipid metabolism in hepatocytes and how this may impact alcohol-induced steatosis. Our novel studies demonstrate that scaffolding of PKA by AKAP12 sustains PKA activation and promotes phosphorylation of PKA’s substrates involved in fatty acid biosynthesis and oxidation, which potently reduces steatosis and inflammation in the liver during ALD.

## Results

### Identification of AKAP12’s interactome in hepatocytes from alcohol-fed mice

To identify the interacting partners of AKAP12 in hepatocytes, crosslinking proteomics was performed in hepatocytes from pair-fed or alcohol-fed mice. AKAP12’s interactome included PKA subunits, MAPK family players, ADRB2, and known substrates of PKA-dependent phosphorylation, ACACA or Acetyl CoA carboxylase 1 (ACC1), pyruvate dehydrogenase (PDHA1) and PNPLA2 (adipose triglyceride lipase (ATGL))^6^ (Table 1). Other interactions of AKAP12 identified were phosphatases and Rab proteins (Table 1, Data S1).

**Table 1:** Identification of AKAP12-interacting kinases and PKA targets in hepatocytes isolated from pair-fed or ethanol-fed mice

Uniprot ID	Protein name	Gene names	Normalized Intensity CON1	Normalized Intensity CON2	Normalized Intensity ETOH1	Normalized Intensity ETOH2	Average CON	Average ETOH	ETOH/CON-FC
Q5SW9	Acetyl-CoA carboxylase 1	*Acaca*	14,523,333	9,723,769	3,591,119	3,664,896	12,123,551	3,628,008	0.29
P12367	Protein kinase type II-alpha	*Prkar2a*	6,948,980	388,047	3,911,721	261,102	3,668,514	2,086,412	0.57
P18762	Beta-2 adrenergic receptor	*Adrb2*	29,166,042	109,652,492	27,996,084	50,830,462	69,409,267	39,413,273	0.57
P35486	Pyruvate dehydrogenase E1 component subunit alpha	*Pdha1*	18,728,514	61,763,185	11,653,508	19,575,263	40,245,850	15,614,386	0.39
Q63932	Dual specificity mitogen-activated protein kinase kinase 2	*Map2k2*	775,192	0	1,244,718	0	387,596	622,359	1.61
Q9DBC7	Protein kinase type I-alpha regulatory subunit	*Prkar1a*	17,158,928	1,213,412	1,301,303	2,709,497	9,186,170	2,005,400	0.22
Q8BJ56	Patatin-like phospholipase domain-containing protein 2	*Pnpla2*	1,453,202	1,930,883	5,194,876	1,850,961	1,692,042	3,522,918	0.48

AKAP12 crosslinking and mass spectrometry for interaction partners were performed as in methods. The data represents the label-free quantification intensity of AKAP12-interacting kinases and PKA targets normalized to their total expression. Raw data for crosslinking proteomics is shown in Data S1. Proteins of relative quantitation were divided into two categories. Statistical significance was defined as *p* < 0.05 with FC cutoff of 1.5 for up-regulation and 0.67 for down-regulation

*CON* control, *ETOH* ethanol

### AKAP12-PKA scaffolding is reduced during alcohol exposure

**Fig. 1:**
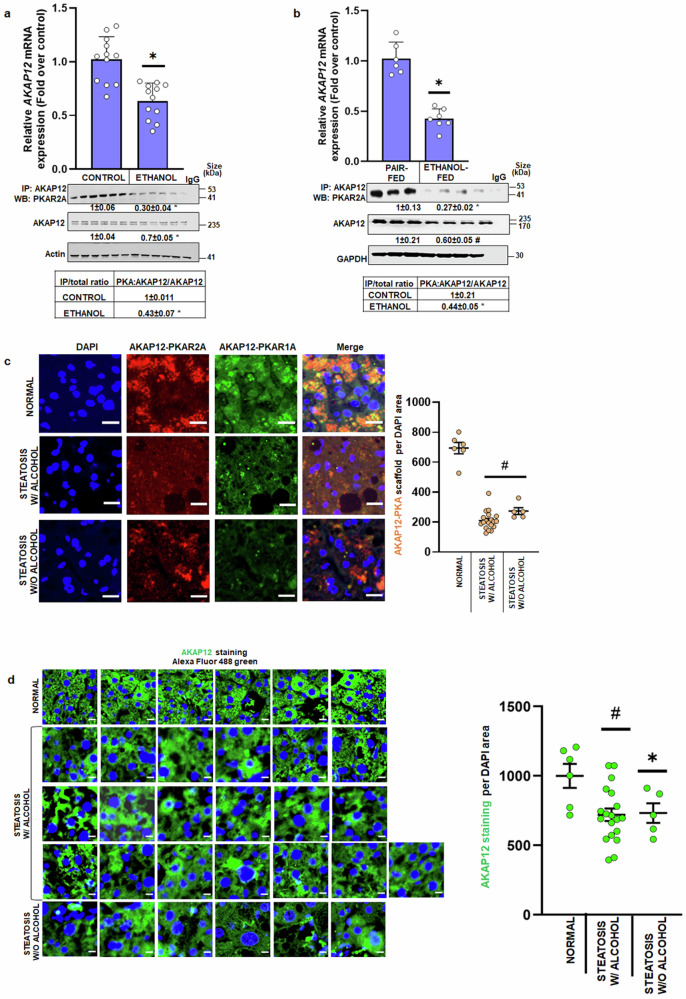
AKAP12-PKA scaffolding is reduced during alcohol exposure. **a** Human hepatocytes were treated with 100 mM ethanol for 24 h. Top panel: *AKAP12* or *HPRT1* (control gene) was quantified by real-time RT-PCR. Data are relative mRNA levels represented as fold over control. *N* = 12, *p < 0.001 vs. control. Bottom panel: Total protein from control or ethanol-treated hepatocytes was co-immunoprecipitated with AKAP12 antibody and western blotted to detect PKAR2A with actin as a loading control. Actin-normalized densitometric quantification is mean ± SE from six experiments. *p < 0.001 vs. control. Uncropped blots with markers are shown in Supplementary Fig. 13. **b** Livers were examined from mice fed alcohol or control diets as described under methods. Top panel: *Akap12* or *Gapdh* (control gene) was quantified by real-time RT-PCR. Data are relative mRNA levels represented as fold over pair-fed from six pair-fed and seven ethanol-fed samples. *p < 0.001 vs. control. Bottom panel: Total liver protein from pair-fed (control) or ethanol-fed mice was co-immunoprecipitated with AKAP12 antibody and western blotted to detect PKAR2 with GAPDH as the loading control. GAPDH-normalized densitometric quantification is mean ± SE from three pair-fed and four ethanol-fed experiments. *p < 0.001 vs. pair-fed, #*p* < 0.05 vs. pair-fed. Uncropped blots with markers are shown in Supplementary Fig. 14. **c** Human liver steatosis tissue array containing 6 normals, 19 steatosis with alcohol, and 5 steatosis without alcohol tissues was stained with PLA probes to detect interactions between AKAP12-PKAR2 (red) and AKAP12-PKAR1 (green). Merged areas between the interacting complexes were quantified by ImageJ. Results are represented as the AKAP12-PKA count per DAPI area. #*p* < 0.001 vs. normal, scale bar=30 µM, and 400× magnification. **d** Human liver steatosis array containing 6 normals, 19 steatosis W/ alcohol, and 5 steatosis W/O alcohol tissues was stained with AKAP12 antibody and probed with Alexa Fluor 488 green probe. Results are represented as the AKAP12 count per DAPI area. #*p* < 0.001, *p < 0.05 vs. normal The tissue array staining and imaging at 200× magnification is shown, scale bar: 20 µm

Our crosslinking proteomics demonstrated that AKAP12’s interaction with PKA’s regulatory subunits was reduced by alcohol exposure (Table 1). To confirm the proteomics analysis, co-immunoprecipitation experiments were performed. Co-immunoprecipitation of PKAR2A with AKAP12 was reduced by 70% in ethanol-treated hepatocytes compared to control (Fig. 1a). This was associated with a 30–40% decrease in total *AKAP12* expression resulting in a 56% decrease in the co-immunoprecipitated AKAP12-PKAR2A/total AKAP12 ratio compared to control (Fig. 1a). Albumin expression in hepatocytes was unchanged after 24-h alcohol exposure (Supplementary Fig. 1). Alcohol-fed mice livers exhibited a 50% suppression of the AKAP12-PKAR2A/total AKAP12 ratio compared to pair-fed mice (Fig.1b). To detect AKAP12’s interaction with PKAR2A or PKAR1A in human liver, a human steatosis tissue array containing 19 steatosis with alcohol use, 5 steatosis without alcohol use, and 6 normal liver tissues was stained with PLA probes. The interaction between AKAP12 and PKAR2A and R1A subunits was inhibited by 70% in human liver steatosis with or without alcohol compared to normal (Fig. 1c, Supplementary Fig. 2). This was associated with a 30% decrease in AKAP12 expression in steatotic tissue compared to normal (Fig. 1d). AKAP12 expression was checked further in a STAM™ model, a MASLD/diabetes model that progresses from steatosis to HCC and is representative of patient populations in this setting. AKAP12 staining and protein expression were reduced in steatosis, MASH, and fibrosis, and dramatically suppressed in HCC stage (Supplementary Fig. 3).

### MicroRNAs regulate the expression of *AKAP12* mRNA during alcohol exposure

**Fig. 2:**
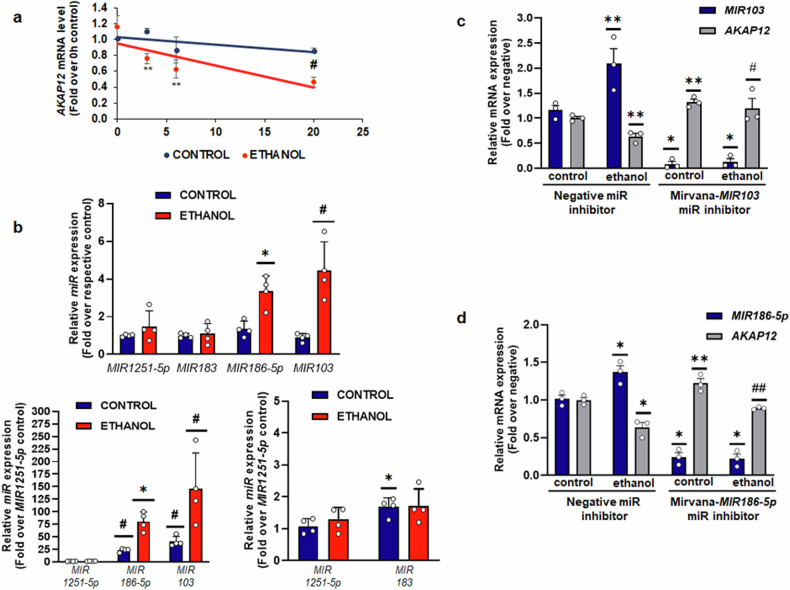
MicroRNAs regulate the expression of *AKAP12* mRNA during alcohol exposure. **a** Actinomycin D chase assays of control or ethanol-treated human hepatocytes was performed as described under methods. Real-time RT-PCR of RNA at each time point detected the relative expression of *AKAP12* mRNA in ethanol-treated samples compared to control. *HPRT1* was used as a normalizing control. Data represent the *AKAP12* mRNA level as fold over 0 h control from three experiments. **P < 0.05, #*p* < 0.01 vs. control. **b** Expression of *MIR103, MIR186-5p*, *MIR183,* and *MIR1251-5p* in control or ethanol-treated hepatocytes was measured by real-time PCR as described in methods. Top graph represents the miR relative expression represented as fold over respective control from four experiments. *P < 0.05, #*p* < 0.001 vs. control. Bottom graph represents miR expression relative to *MIR1251-5p* in control cells from four experiments. #*p* < 0.001, *p < 0.05 vs. mir1251-5p control. **c** Cells were treated with mirvana MIR negative control or *MIR103* inhibitors in the absence or presence of ethanol as described in methods. Data are relative *MIR103* or *AKAP12* mRNA levels represented as fold over negative control from three experiments. **P < 0.01, *p < 0.001 vs. negative miR+control, #*p* < 0.05 vs. negative miR+ethanol. **d** Cells were treated with mirvana MIR negative control or *MIR186-5p* inhibitors in the absence or presence of ethanol as described in methods. Data are relative *MIR186-5p* or *AKAP12* mRNA levels represented as fold over negative control from three experiments. *p < 0.01, **p < 0.05 vs. negative miR, ##*p* < 0.05 vs. negative miR+ethanol, or mirvana mir186-5p

Since the total expression of *AKAP12* mRNA was reduced by alcohol exposure in vitro and in vivo (Fig. 1), we evaluated putative mechanisms for this effect. Bulk RNA sequencing and transcriptomics evaluation from two publicly available datasets (GEP liver atlas and GEO [GDS4389]) showed decreased *AKAP12* expression in human ALD, MASLD, and alcohol-associated hepatitis (Supplementary Fig. 4a, b). Publicly available single-cell RNA sequencing from the GEPliver database^33^ revealed expression of AKAP12 in different cell types of the liver (Supplementary Fig. 4c, f, g) with a decrease in expression observed in hepatocytes, cholangiocytes and monocytes during MASLD and advanced cirrhosis and a decrease in endothelial cell expression during cirrhosis (Supplementary Fig. 4d, e, f, g). Alcohol did not alter the transcriptional activity of *AKAP12* promoter (Supplementary Fig. 5) but decreased the stability of its mRNA compared to control in actinomycin D chase assays (T1/2 control~57 h, T1/2 ethanol ~17 h) (Fig. 2a). *AKAP12* is known to be targeted by microRNAs, *MIR186*, *MIR183*, *MIR1251-5p*, and *MIR103* during liver cirrhosis and HCC.^31,34,35^ Out of the known *AKAP12 MIRs*, alcohol induced the expression of *MIR103* and *MIR186-5p* by 3 to 4-fold compared to control hepatocytes (Fig. 2b, top panel). *MIR183* and *MIR1251-5p* expression remained unaffected by alcohol exposure (Fig. 2b, top panel). The basal expression of *MIRs 103 and 186-5p* was 25–40 fold higher than *MIR1251-5p* (Fig. 2b, bottom panel) and exhibited further increase by alcohol (Fig. 2b, bottom panel). *MIR183* expression was 1.5-fold higher than *MIR1251-5p* in hepatocytes and neither was changed by alcohol (Fig. 2b, bottom panel). *MIR* inhibitor, mirvana-103 caused a 90% reduction in *MIR103* expression and recovered the expression of *AKAP12* mRNA suppressed by alcohol (Fig. 2c). The *MIR186* inhibitor, Mirvana-186 reduced *MIR186-5p* by 80% compared to negative control and recovered alcohol-suppressed *AKAP12* mRNA levels (Fig. 2d). The data suggests that induction of these *MIRs* may be responsible for *AKAP12* mRNA destabilization upon alcohol exposure.

### Modulation of *AKAP12* expression regulates PKA activity, triglyceride content, and lipid accumulation in hepatocytes

**Fig. 3:**
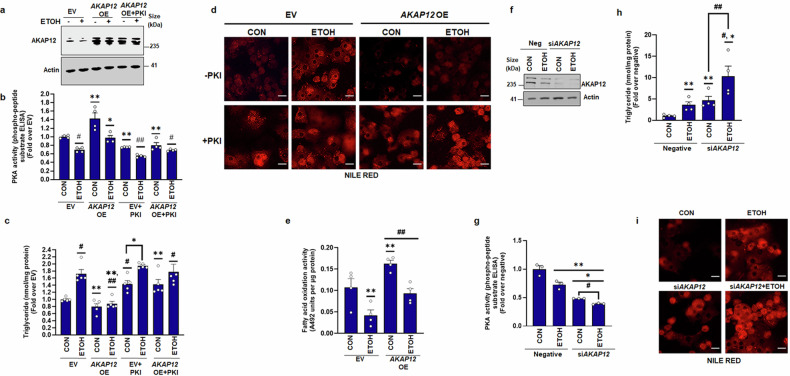
Modulation of *AKAP12* expression regulates PKA activity, triglyceride content, and lipid accumulation in hepatocytes. Human hepatocytes were transfected with EV or *AKAP12* vector with or without PKI under control (CON) or ethanol (ETOH) conditions as described under methods. **a** Overexpression of AKAP12 was assessed by western blotting using actin as a loading control. Uncropped blots with markers are shown in Supplementary Fig. 15. **b** Total protein extracts were assayed for PKA activity using a phospho-peptide ELISA as described under methods. Data represents the PKA activity as fold over EV from four experiments. **p < 0.001, #*p* < 0.05 vs. EV, *p < 0.05 vs. EV + ETOH, ##*p* < 0.001 vs. EV, or EV + PKI. **c** Triglycerides extracted from hepatocytes and standard triglycerides of known concentration were quantified using the triglyceride-Glo™ kit. The data represents nmoles of triglyceride per mg protein expressed as fold over EV from 5 experiments. **p < 0.05 vs. EV, #*p* < 0.01 vs. EV, ##*p* < 0.01 vs. EV + ETOH, *p < 0.05 vs. EV + PKI. **d** Hepatocyte lipids were stained by Nile red staining as described in “Methods”. A representative staining from three experiments is shown. 40 µM scale bar, 400× magnification. **e** FAO activity was measured as described in “Methods”. The data represent FAO activity (A492 absorbance units per ug protein) from four experiments. **p < 0.05 vs. EV, ##*p* < 0.05 vs. EV + ETOH. **f** Human hepatocytes were transfected with negative control or *AKAP12* siRNA as described under methods. The silencing of *AKAP12* was confirmed by AKAP12 western blotting. Uncropped blots with markers are shown in Supplementary Fig. 16. **g** Total protein extracts were assayed for PKA activity using a phospho-peptide ELISA as described under methods. Data represents the PKA activity as fold over EV from three experiments. **P < 0.01 vs. negative, *p < 0.01 vs. negative+ETOH, #*p* < 0.001 vs. si*AKAP12*. **h** Hepatocyte triglycerides were measured as in ''**c**'' above. The data represent nmoles of triglyceride per mg protein expressed as fold over negative. **p < 0.05, #*p* < 0.01 vs. negative, *p < 0.05 vs. negative+ETOH, ##*p* < 0.05 vs. siAKAP12. **i** Hepatocyte lipids were stained with Nile red as in ''**d**'' above. A representative staining from three experiments is shown. 40 µM scale bar, 400× magnification

To examine whether AKAP12 could modulate PKA functionality, triglyceride content, and lipid accumulation, we altered *AKAP12* expression in hepatocytes by transfection of the *AKAP12* vector. We checked the transfection of this vector in human hepatocytes by western blotting for AKAP12 protein overexpression (Fig. 3a). Alcohol inhibited PKA activity on a phospho-peptide substrate by 40% compared to control (Fig. 3b). This was associated with a 1.7-fold induction in triglyceride levels compared to control (Fig. 3c). *AKAP12* overexpression induced both basal and alcohol-suppressed PKA activity (Fig. 3b) and suppressed basal and alcohol-stimulated triglyceride content (Fig. 3c). To evaluate whether PKA activation was required for AKAP12 to exert its influence on lipid accumulation, we suppressed PKA activity by the protein kinase inhibitor peptide (PKI), a specific PKA inhibitor that caused a 30–35% decrease in PKA activity compared to EV (control) (Fig. 3b). PKI increased triglyceride content in hepatocytes by 1.5-fold compared to EV (control) and further increased ethanol-enhanced triglyceride levels (Fig. 3c). *AKAP12* overexpression under PKI conditions did not suppress basal or alcohol-enhanced triglycerides (Fig. 3c). Like the triglyceride content, *AKAP12* overexpression suppressed both basal and alcohol-stimulated lipid staining (nile red) whereas PKA inhibition prevented AKAP12 from suppressing lipid accumulation (Fig. 3d). These data suggest that AKAP12 suppresses triglyceride content and lipid accumulation in hepatocytes in a PKA-dependent manner. AKAP12 stimulated both basal and alcohol-suppressed FAO in human hepatocytes (Fig. 3e). Silencing *AKAP12* in human hepatocytes (Fig. 3f) suppressed basal PKA activity and further reduced alcohol-suppressed PKA activation compared to negative control (Fig. 3g). *AKAP12* silencing potentiated both basal and alcohol-stimulated triglyceride levels (Fig. 3h) and lipid accumulation (Fig. 3i).

### Inhibition of AKAP12-PKA interaction suppresses PKA activity and induces lipid accumulation in hepatocytes

**Fig. 4:**
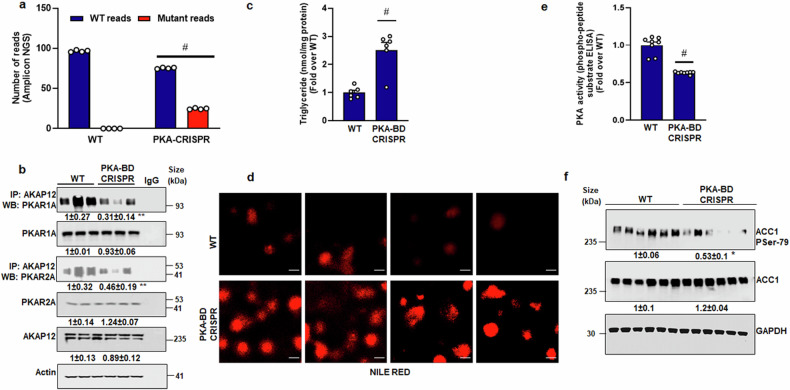
Inhibition of AKAP12-PKA interaction suppresses PKA activity and induces lipid accumulation in hepatocytes. Human hepatocytes were transfected with CRISPR reagents and SaCas9 vector to cause CRISPR-directed deletion of AKAP12’s PKA-BD as described under methods. **a** Next-generation amplicon sequencing of genomic DNA from WT or CRISPR-edited cells was performed. The number of WT or mutant reads from each condition is represented as percentage of total reads from four experiments. #*p* < 0.001 vs. WT. **b** Total protein from WT or CRISPR-edited cells was co-immunoprecipitated with AKAP12 antibody and western blotted to detect PKAR1 or PKAR2. Actin was used as a loading control. Actin-normalized densitometric quantification from six experiments is shown. **p < 0.05 vs. WT Uncropped blots with markers are shown in Supplementary Fig. 17. **c** Triglycerides were quantified as described under “Methods”. The data represent nmoles of triglyceride per mg protein expressed as fold over WT. #*p* < 0.001 vs. WT. **d** Hepatocyte lipids were staine**d** by Nile red staining as described in “Methods”. *N* = 4, 40 µM scale bar, 400× magnification. **e** Total protein extracts were assayed for PKA activity using a phospho-peptide ELISA as described under methods. Data represent the PKA activity as fold over EV from eight experiments. #*p* < 0.001 vs. WT. **f** Total protein from WT or CRISPR cells was western blotted for P-ACC1 or ACC1 protein expression. Data normalized to GAPDH (loading control) is mean ± SE from six experiments. *p < 0.01 vs. WT. Uncropped blots with markers are shown in Supplementary Fig. 18

AKAP12 possesses a C-terminal 1526–1780 residue PKA binding domain region denoted as PKA-BD.^36,37^ To examine the influence of AKAP12-PKA interaction on PKA activation and lipid accumulation, CRISPR-directed gene editing was performed to delete the PKA-BD of AKAP12 in hepatocytes. Next-generation sequencing revealed that 25% of genomic DNA-derived amplicons from PKA-BD CRISPR cells contained a deletion of the DNA region corresponding to this domain (Fig. 4a). PKA-BD CRISPR suppressed AKAP12’s interaction with PKAR1A by 70% and with PKAR2A by 60% compared to control (WT) cells (Fig. 4b). AKAP12’s PKA-BD CRISPR induced triglyceride content in hepatocytes by 2.5-fold compared to WT cells (Fig. 4c). This was associated with increased lipid staining in hepatocytes (Fig. 4d). AKAP12 PKA-BD CRISPR suppressed PKA activity by 40% compared to WT (Fig. 4e). ACC1, a substrate of PKA and AMPK whose activity is inhibited by phosphorylation,^7^ was identified as an interacting partner of AKAP12 in proteomics analysis (Table 1). We tested whether AKAP12 PKA-BD deletion regulated ACC1 activation. PKA-BD CRISPR suppressed ACC1 phosphorylation at the inhibitory phosphorylation site, Ser79, by 50% compared to WT (Fig. 4f).

### Forced expression of *AKAP12* regulates lipid accumulation in a mouse model of ALD

**Fig. 5:**
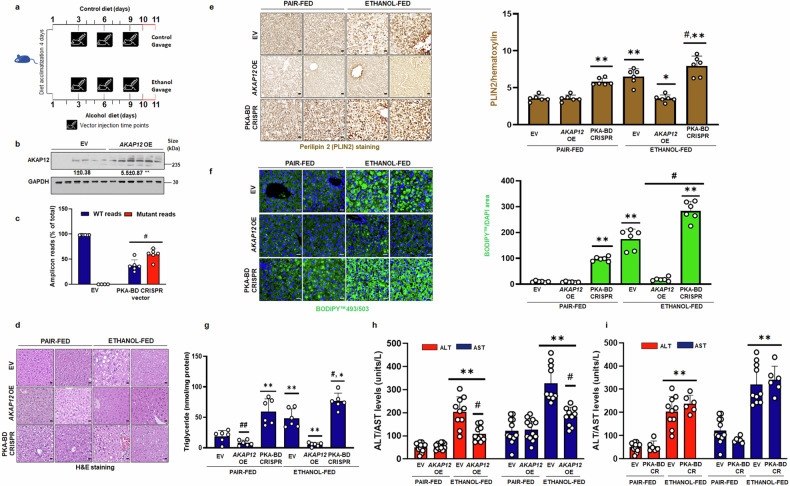
Modulation of total *AKAP12* expression or AKAP12-PKA binding domain regulates lipid accumulation in a mouse model of ALD. **a** Mice were injected with *AKAP12*, PKA-BD CRISPR vector or control vector (EV) at day 3, day 6, and day 9 of control or ethanol-diet feeding as described in methods. **b** *AKAP12* overexpression was assessed by western blot of AKAP12 from control or *AKAP12* vector injected livers. Data represented as GAPDH-normalized densitometry is fold over EV from six experiments. *p < 0.001 vs. EV. Uncropped blots with markers are shown in Supplementary Fig. 19. **c** Next-generation amplicon sequencing of genomic DNA from control or PKA-BD CRISPR vector-treated livers was performed. The number of WT or CRISPR edited reads from each condition is represented as the percentage of total reads from four experiments. #*p* < 0.001 vs. WT. **d** H&E staining, 20 µM scale bar, 200× magnification. **e** PLIN2 staining was performed as described under methods. ImageJ quantification of the PLIN2 per hematoxylin is representative of six experiments. **p < 0.001 vs. EV+pair-fed, #*p* < 0.001 vs. EV+ethanol, *p < 0.05 vs. EV+ethanol. 20 µM scale bar, 200× magnification. **f** BODIPY™ staining was performed as described under methods. BODIPY count per DAPI area is shown from six experiments. Two representative stains out of six tissues are shown. **p < 0.001 vs. EV+pair-fed, #*p* < 0.001 vs. EV+ethanol, *p < 0.05 vs. EV+ethanol. 20 µM scale bar, 200× magnification. **g** Triglycerides were quantified as described under methods. The data represent nmoles of triglyceride per mg protein from six experiments. **p < 0.01 vs. EV+pair-fed, #*p* < 0.001 vs. EV+pair-fed, *p < 0.01 vs. EV+ethanol. **h** Serum liver injury markers, ALT and AST were measured in *AKAP12* overexpression livers using the ALT/AST assay reagents following manufacturer’s protocol described in methods. Data represent the ALT or AST activity units/L from 6 to 12 experiments. **p < 0.001 vs. pair-fed, #*p* < 0.001 vs. ethanol-fed. **i** ALT and AST levels were measured in PKA-BD CRISPR livers as in ''**h**'' above. **p < 0.001 vs. pair-fed

The effect of AKAP12 modulation on liver injury, triglyceride accumulation, and steatosis were evaluated in control or ethanol-fed mice injected with EV or an *AKAP12* overexpression vector according to the plan in Fig. 5a. *AKAP12* vector injection caused a 4.5-fold induction in AKAP12 protein in livers compared to EV (Fig. 5b). Livers transfected with the *AKAP12*, or control vector (EV) were detected by AKAP12 co-localization with the hepatocyte marker, albumin, HSC marker, desmin, or endothelial cell marker, CD32B. Endogenous AKAP12 in EV tissues co-localized with albumin, desmin, and CD32B (Supplementary Fig. 6). *AKAP12* overexpression caused a 5.5-fold induction of AKAP12 in albumin-positive areas and a 2-fold induction in desmin or CD32B-positive areas compared to EV (Supplementary Fig. 6). In *AKAP12* overexpressed livers, 77% of hepatocytes stained AKAP12, with 4% staining in HSCs and 18% staining in endothelial cells (Supplementary Fig. 6). Alcohol feeding increased lipid droplets in the liver compared to pair-fed livers as judged by H&E (Fig. 5d), lipid droplet marker, perilipin-2 (PLIN2) expression (Fig. 5e) or BODIPY™ lipid staining (Fig. 5f) and *AKAP12* overexpression suppressed these parameters (Fig. 5d–f). *AKAP12* overexpression dramatically reduced triglyceride content both under basal and ethanol-fed conditions by 60–70% compared to pair-fed controls (Fig. 5g). *AKAP12* overexpression reduced ethanol-potentiated liver injury by 40–50% compared to EV+ethanol-fed group but did not completely normalize it to EV levels (Fig. 5h) *AKAP12* overexpression did not alter parameters of alcohol metabolism, CYP2E1 expression or activity in the liver (Supplementary Fig. 7).

### Disruption of AKAP12’s PKA-binding region potentiates lipid accumulation in vivo

The effect of gene-editing the PKA-binding domain of AKAP12 on liver injury, triglyceride accumulation, and steatosis was assessed in control or ethanol-fed mice injected with AKAP12 PKA-BD CRISPR-Cas9 deletion vector containing a SaCas9 enzyme controlled by a hepatocyte-specific thyroxin binding globulin (TBG) promoter (Fig. 5a). Next-generation sequencing revealed that 60% of the genomic DNA-derived amplicons from CRISPR-edited livers contained a deletion of the DNA region corresponding to AKAP12’s PKA-BD (Fig. 5c). The specificity and efficiency of transfection of hepatocyte-specific PKA-BD CRISPR was assessed by co-staining of the CRISPR enzyme, SaCas9 with hepatocyte marker, albumin or HSC marker, desmin. The TBG-driven SaCas9 enzyme strongly co-localized with albumin-positive hepatocytes in PKA-BD CRISPR samples but no-colocalization was observed with desmin-positive HSCs (Supplementary Fig. 8). CRISPR-transfected SaCas9 enzyme co-localized with BODIPY-positive cells both in PKA-BD CRISPR and PKA-BD CRISPR+ ethanol livers, indicating positive AKAP12 PKA-BD CRISPR modulation in lipid-stained cells (Supplementary Fig. 9). Basal or alcohol-potentiated lipid droplet PLIN2 expression (Fig. 5e) or BODIPY™ lipid staining (Fig. 5f) was enhanced by PKA-BD-CRISPR. PKA-BD CRISPR dramatically induced triglyceride content both under basal and ethanol-fed conditions by 3-4-fold compared to EV+pair-fed mice (Fig. 5g). PKA-BD CRISPR+ethanol group exhibited a 1.6-fold increase in triglycerides compared to EV+ethanol-fed group (Fig. 5g). Ethanol-feeding alone or in combination with PKA-BD-CRISPR increased ALT and AST levels (Fig. 5i) by 3.2–4 fold compared to pair-fed controls. PKA-BD-CRISPR did not alter CYP2E1 activity or expression in the liver (Supplementary Fig. 10).

### AKAP12 modulation alters PKA’s lipogenic targets in mouse liver

**Fig. 6:**
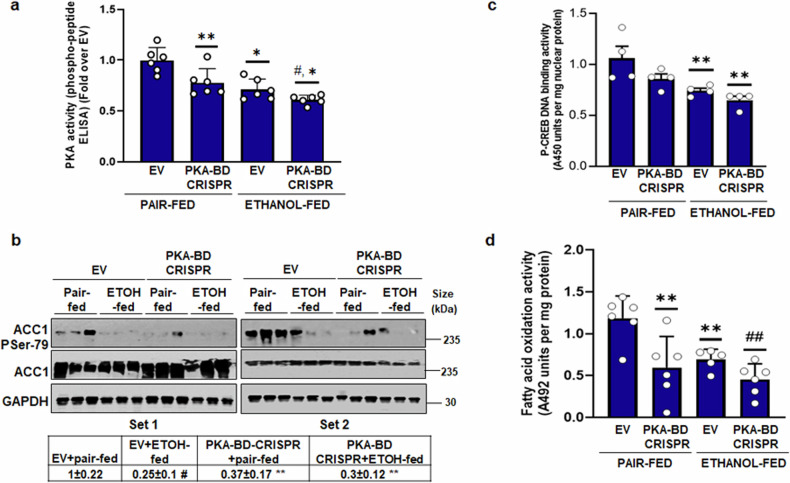
AKAP12 PKA-BD CRISPR alters PKA’s lipogenic targets in mouse liver. Mice were injected with CRISPR vectors as described in Fig. 5A legend. **a** Liver extracts were assayed for PKA activity using a phospho-peptide ELISA as described under methods. Data represents the PKA activity as fold over EV from six experiments**p < 0.05, *p < 0.01 vs. EV+pair-fed, #*p* < 0.05 vs. EV+ethanol. **b** Liver protein was western blotted to probe for P-ACC1, total ACC1 or GAPDH (loading control). Data represented as GAPDH-normalized densitometry is fold over EV+pair-fed from six experiments. #*p* < 0.01, **p < 0.05 vs. EV+pair-fed. Uncropped blots with markers are shown in Supplementary Fig. 20. **c** Nuclear extracts prepared from the liver were processed to measure P-CREB DNA binding activity as described under methods. Data are represented as the A450 absorbance units per mg of nuclear protein from four experiments. **p < 0.05 vs. EV+pair-fed. **d** FAO activity in total extracts was measured as described in “Methods”. The data represent FAO activity (A492 absorbance units per mg liver protein) from five to six experiments. **p < 0.05 vs. EV+pair-fed, ##*p* < 0.05 vs. EV+ethanol

To examine the effect of AKAP12 modulation on PKA’s lipogenic targets, we performed PKA-BD CRISPR or *AKAP12* overexpression experiments in mouse liver. PKA-BD CRISPR of AKAP12 suppressed PKA activity by 25% compared to control livers and decreased ethanol-suppressed PKA activity by 40% compared to control (Fig. 6a). PKA-BD CRISPR suppressed inhibitory phosphorylation of ACC1 both under basal and ethanol-fed conditions by 70% compared to control group (Fig. 6b). Ser133-phosphorylation of CREB by PKA enhances its DNA binding activity on cAMP response elements (CRE) of genes.^6^ The CRE element binding activity of CREB detected by an antibody for S133-CREB was reduced by 15% in PKA-BD CRISPR modified livers compared to control but did not achieve statistical significance (Fig. 6c). Ethanol suppressed P-CREB activity by 26% compared to control and PKA-BD CRISPR significantly reduced ethanol-mediated suppression of P-CREB activity by 35% compared to control (Fig. 6c). FAO activity in the liver was suppressed by PKA-BD-CRISPR both under basal and ethanol-fed conditions (Fig. 6d). Overexpression of *AKAP12* in mouse liver reversed the suppressive effect of ethanol on PKA activity, P-CREB activation and FAO (Supplementary Fig. 11a, b, c). Basal induction of PKA activity and P-CREB activation was also observed in normal liver over-expressing *AKAP12* (Supplementary Fig. 11a, b).

### STK25 is involved in alcohol’s disruptive effect on the AKAP12-PKA scaffold

**Fig. 7:**
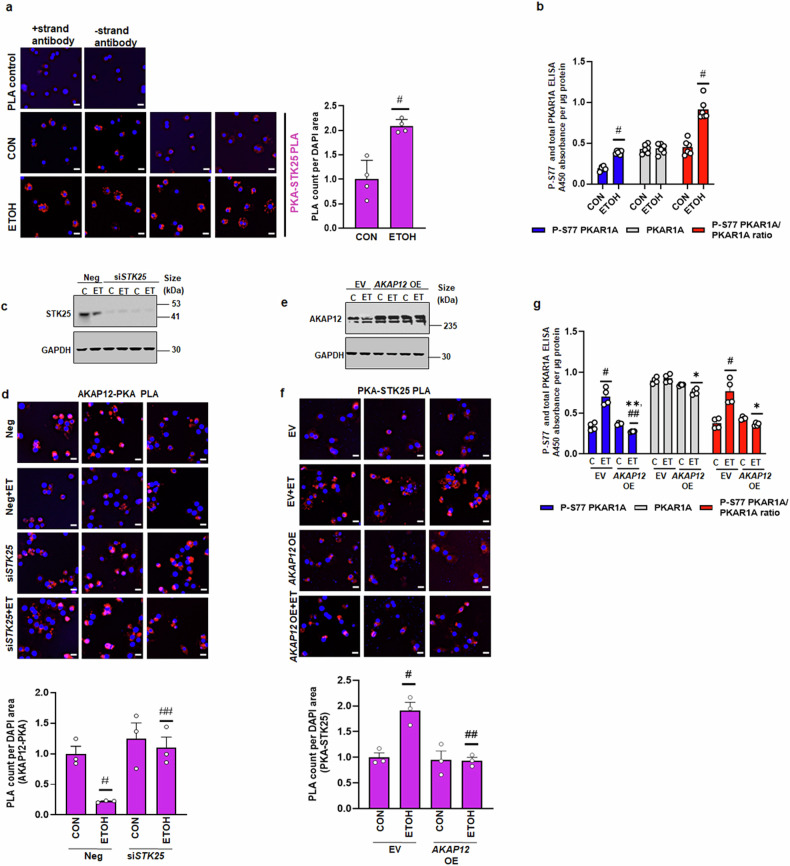
STK25 is involved in alcohol’s disruptive effect on the AKAP12-PKA scaffold. **a** Hepatocytes under control or ethanol conditions were subjected to PLA staining to detect the interaction between PKAR1A and STK25 as described in methods. PLA staining of PKAR1A-STK25 interaction (red) and nuclear DAPI (blue) was quantified by ImageJ and represented as the PLA count per DAPI area from four experiments. #*p* < 0.001 vs. control. 20 µM scale bar, 200× magnification. **b** Control or ethanol-treated hepatocytes in 96-well plates were subjected to cell-based ELISA assay as described in methods to detect phospho-S77-PKAR1A or PKAR1A using antibodies. The data represents the A450 nm absorbance per µg of cellular protein and the ratio of phospho-S77/total PKAR1A from six experiments. #*p* < 0.001 vs. control. **c** Hepatocytes were transfected with a negative control or *STK25* siRNA in the absence or presence of ethanol as described under methods. Western blot of STK25 or GAPDH control was performed to determine the silencing efficiency. Uncropped blots with markers are shown in Supplementary Fig. 21. **d** PLA staining of AKAP12-PKA (red) and nuclear DAPI (blue) interaction under the conditions in “**c**” above was quantified by ImageJ and represented as the PLA count per DAPI area from three experiments. #*p* < 0.05 vs. negative, ##*p* < 0.5 vs. negative+ethanol. 20 µM scale bar, 200× magnification. **e** Hepatocytes were transfected with EV or *AKAP12* vector in the absence or presence of ethanol as described under methods. Western blot of AKAP12 or GAPDH control was performed to determine the overexpression efficiency. Uncropped blots with markers are shown in Supplementary Fig. 21. **f** PLA staining of PKA-STK25 interaction (red) and nuclear DAPI (blue) under the conditions in “**e**“ above was quantified by ImageJ and represented as the PLA count per DAPI area from three experiments. *N* = 3, #*p* < 0.01 vs. EV, ##*p* < 0.01 vs. EV+ethanol. 20 µM scale bar, 200× magnification. **g** Control or ethanol-treated hepatocytes transfected with *AKAP12* vector or EV were subjected to cell-based ELISA as described in legend for “**b**“ above. The data represents the A450 nm absorbance per µg of cellular protein and the ratio of phospho-S77/total PKAR1A from four experiments. #*p* < 0.01 vs. EV, **p < 0.05 vs. EV, ##*p* < 0.001 vs. EV+ethanol, *p < 0.01 vs. EV or EV+ethanol

It has been demonstrated that the stress-associated kinase STK25 phosphorylates the PKAR1A regulatory subunit on S77 and S83, hence inhibiting PKA function in cardiomyocytes.^38^ We investigated whether alcohol affected the interaction between STK25 and PKAR1A as well as STK25 activity. Our findings showed that alcohol exposure in hepatocytes doubled the PKAR1A-STK25 interaction in comparison to the control group without changing the total amounts of STK25 (Fig. 7a). ELISA-based assays of control and ethanol-treated hepatocytes with the PKAR1A-S77 phospho-antibody (a marker of STK25 activity) revealed that alcohol raised S77-PKAR1A phosphorylation by 2.2-fold and the S77-PKAR1A/PKAR1A ratio by 3-fold compared to control (Fig. 7b). In hepatocytes, we also noticed a faint interaction between AKAP12 and STK25; however, alcohol did not appear to alter this interaction (Supplementary Fig. 12).

To investigate the potential role of STK25 in modulating the AKAP12-PKAR1A scaffold, we silenced *STK25* under control or ethanol-treated conditions (Fig. 7c). Alcohol-dependent suppression of AKAP12-PKAR1A scaffolding was recovered by silencing *STK25* (Fig. 7d). We further tested whether AKAP12 could modulate the alcohol-potentiated PKAR1A-STK25 scaffold. Indeed, we observed that forced expression of *AKAP12* (Fig. 7e) normalized the increased STK25-PKAR1A interaction in hepatocytes (Fig.7f). Further, we observed that *AKAP12* forced expression normalized alcohol-potentiated S77-PKAR1A phosphorylation (or STK25 activity) in hepatocytes (Fig. 7g) without causing a change in PKAR1A total levels (Fig. 7g).

### Consequences of AKAP12 modulation on hepatocytes and total liver

**Fig. 8:**
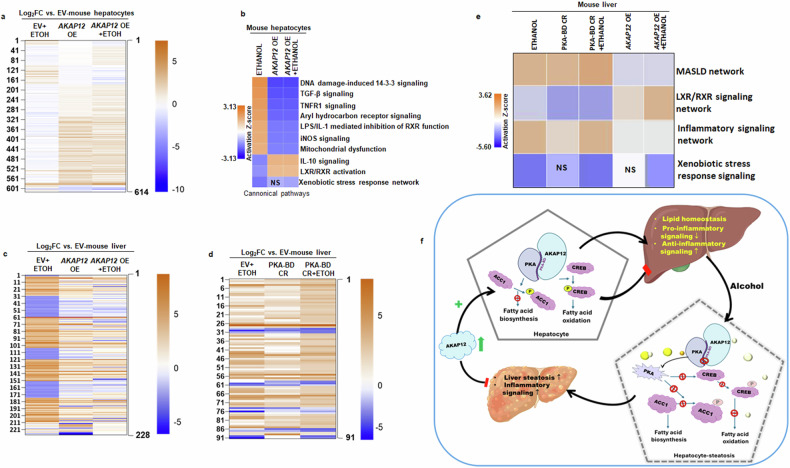
Consequences of AKAP12 modulation on hepatocytes and total liver. **a** Gene heatmap of DEG RNA seq analysis for hepatocytes from EV, EV+ethanol (ETOH), *AKAP12* overexpression (*AKAP12* OE) or *AKAP12* OE + ETOH mice. The 614 gene set is listed in Data S2 under the “significant pathways” tab. **b** IPA pathway analysis of genes from “**a**“ above. **c** Gene heatmap DEG RNA seq analysis of total liver from EV, EV + ETOH, *AKAP12* OE, or *AKAP12* OE + EOH mice. The 228 gene set is listed in Data S3 under the “significant pathways AKAP12 OE” tab **d** Gene heatmap DEG RNA seq analysis for total liver from EV, EV + ETOH, PKA-BD CRISPR (PKA-BD CR), or PKA-BD CR + ETOH mice. The 91 gene set is listed in Data S3 under the “significant pathways PKABD CR” tab. **e** IPA analysis of pathways from “**c**“ and “**d**“ above. Significant DEGs are shown in Data S2 and 3. **f** Summary of findings. Parts of this figure are generated by BioRender (https://www.biorender.com). AKAP12, a kinase anchoring protein, interacts with the PKA-binding domain (PKA-BD) of PKA and maintains PKA activation in hepatocytes. Inhibition of the AKAP12-PKA scaffold by alcohol or by editing the PKA-BD alters the activity of PKA substrates that are controlled by PKA-dependent phosphorylation. ACC1’s inhibitory phosphorylation increased by PKA activation inhibits fatty acid biosynthesis and CREB, a phospho-activated PKA substrate, enhances FAO and negatively regulates inflammatory signaling. Loss of the AKAP12-PKA scaffold thereby potentiates steatosis and inflammation. Increased AKAP12 expression in the liver can overcome steatosis in alcohol-damaged liver and is associated with decreased inflammatory signaling in hepatocytes. Therapeutic strategies to sustain AKAP12-PKA scaffold such as peptide mimetics could be a potential approach targeting alcohol-associated liver disease

To understand the global consequences of *AKAP12* overexpression, bulk RNA sequencing of hepatocytes isolated from EV, EV+ethanol, *AKAP12* overexpression ± ethanol livers was performed (Fig. 8a, Fig. 8b, Data S2). As demonstrated by the heatmap in Fig. 8a, 1145 differentially expressed genes (DEGs) in hepatocytes isolated from liver exhibited a significant change in expression upon alcohol exposure and showed a dramatic regulation by *AKAP12* overexpression both under basal and ethanol-exposed conditions. These RNAs were analyzed by Ingenuity pathway analysis tool (IPA) (Fig. 8b, Data S2). Significant networks dysregulated by alcohol and normalized by *AKAP12* overexpression included inflammatory signaling networks, mitochondrial dysfunction, and LXR/RXR activation network (Fig. 8b, Data S2). These RNAs were also representative of hepatic steatosis and MASLD disease networks (Data S2). Pathways related to xenobiotic stress response that were regulated by alcohol were not altered by *AKAP12* overexpression (Fig. 8b, Data S2). *AKAP12* overexpression did not regulate xenobiotic stress signals under normal conditions (Fig. 8b, Data S2). Total liver from *AKAP12* overexpression samples exhibit normalization of 228 RNAs that were dysregulated by alcohol (Fig. 8c, Data S3). Livers from PKA-BD CRISPR mice exhibited significant modulation of 91 RNAs whose expression was sustained or further dysregulated under alcohol exposure (Fig. 8d, Data S3). IPA analysis revealed that inflammatory networks, LXR/RXR activation, and MASLD networks were dysregulated by PKA-BD CRISPR and normalized by *AKAP12* overexpression (Fig. 8e, Data S3). AKAP12 overexpression at baseline did not affect xenobiotic stress signals and did not alter alcohol’s effect on this pathway in the liver (Fig. 8e, Data S3). The findings summarized in Fig. 8f are discussed.

## Discussion

Since hepatocytes are the main sites of lipid metabolism,^39^ studies investigating the AKAP12-PKA axis as a regulator of lipid homeostasis were conducted in these cells. AKAP12 is a dual specificity anchoring protein that can interact with both RI and R2 regulatory subunits of PKA^40^ which we confirmed by interactome proteomics (Table 1, Data S1). Together with co-immunoprecipitation and PLA assays in mouse liver, human steatotic liver (with or without alcohol use) and hepatocytes, the results demonstrate that alcohol exposure drastically reduces the interaction between AKAP12 and PKA (Fig. 1). A general decline in *AKAP12* expression is observed upon alcohol exposure (Fig. 1) and in non-alcohol mouse models of liver disease progression (Supplementary Fig. 3). In advanced disease states like cirrhosis and HCC, the decline in expression appears more drastic, probably owing to known genetic (DNA methylation) and epigenetic mechanisms (microRNAs) that regulate *AKAP12*.^31^ Human scRNA-Seq data from public domain shows that *AKAP12* is expressed in different cell types of the liver with decreased expression during MASLD with a more dramatic drop in advanced cirrhosis specially in endothelial cells and hepatocytes. Although alcohol did not affect AKAP12 promoter activity (Supplementary Fig. 5), we observed that known AKAP12-targeting microRNAs, *MIR103,* and *MIR183*, induced in ALD^31,41,42^ and in human hepatocytes treated with alcohol (Fig. 2), negatively regulated AKAP12 levels (Fig. 2). Therefore, alcohol-dependent increase in these miRs could be a mechanism of overall decline in AKAP12.

We hypothesized that modulation of AKAP12 reserves regulates PKA activation and lipid accumulation in hepatocytes. In support of this hypothesis, our results demonstrate that forced expression of *AKAP12* could overcome the suppressive effect of alcohol on PKA activity and lipid accumulation, whereas silencing *AKAP12* had the opposite effect (Fig. 3). Inhibition of PKA by PKI prevented AKAP12 from regulating lipid accumulation (Fig. 3), suggesting that AKAP12 acts via PKA activation. Confirming the in vitro results, forced expression of *AKAP12* in the liver of alcohol-fed or pair-fed mice suppressed lipid accumulation and expression of lipid droplet protein, PLIN2 that is induced during alcohol-associated steatosis and positively regulates triglyceride accumulation and lipid droplet numbers^43–45^ (Fig. 5). However, despite the inhibition of steatotic phenotype, liver injury markers were lowered but not completely normalized by *AKAP12* overexpression (Fig. 5h). We noticed that *AKAP12* overexpression did not regulate markers of alcohol metabolism, CYP2E1 (Supplementary Fig. 7). Since CYP2E1 activity is one of the mechanisms of alcohol-potentiated liver injury,^46^ we speculate that the lack of significant effect of AKAP12 modulation on CYP2E1 activity might in part, be responsible for the decrease but not complete normalization, of transaminitis in our model. RNA sequencing analysis has provided further insight into the global consequences of AKAP12 modulation. The fundamental premise of this work is that the crosstalk of PKA with its AKAP, AKAP12 maintains lipid homeostasis in the liver, disruption of which may favor steatotic phenotype. The PKA-BD of AKAP12 is a C-terminal 1526–1780 residue amphipathic alpha helical domain.^36,37^ We assessed if hepatocyte lipid homeostasis could be regulated by interfering with the AKAP12-PKA scaffold. Deleting PKA-BD of AKAP12 by CRISPR gene editing dramatically suppressed AKAP12’s interaction with either PKAR1 or PKAR2 in hepatocytes (Fig. 4). Interfering with the AKAP12-PKA scaffold increased triglyceride content and enhanced lipid accumulation in cultured hepatocytes (Fig. 4) These effects were mirrored in mice with hepatocyte-specific gene editing of AKAP12 PKA-BD (Fig. 5). AKAP12’s interactome in hepatocytes consisted of the lipogenic target of PKA, ACC1 and PDHA^6^ that exhibited suppressed interaction with AKAP12 during alcohol exposure (Table 1). We believe these PKA substrates were pulled down with AKAP12 due to its interaction with PKA and led us to infer that the AKAP12-PKA scaffold may be present at the sites of PKA substrates. Whether this scaffold regulated PKA’s activity on these substrates was evaluated. The PKA target, ACC1 is involved in fatty acid biosynthesis. Loss of ACC1 phosphorylation stimulates de novo lipogenesis during MASLD.^9^ Inhibiting ACC1 activity suppresses hepatic steatosis.^12^ We convincingly demonstrated that AKAP12 PKA-BD disruption suppressed ACC1 phosphorylation indicative of increased ACC1 activity in hepatocytes (Fig. 4), which could be a putative mechanism for increased steatosis observed upon disruption of the AKAP12-PKA scaffold. PKA-dependent activation of the transcription factor CREB at S133 inhibits lipid accumulation, promotes FAO and regulates inflammatory signaling.^13,18^ PKA-BD CRISPR suppressed CREB activation, reducing FAO whereas *AKAP12* overexpression had the reverse effect (Fig. 6, Supplementary Fig. 11). These findings reiterate the role of AKAP12 in sustaining PKA function and support the role of AKAP12-PKA axis as a potent regulator of the lipogenic program in hepatocytes. We observed that suppression of PKA activation by disruption of the AKAP12-PKA scaffold in normal hepatocytes or liver dysregulates lipid homeostasis (Figs. 5, 6). In healthy hepatocytes, AKAP12 interacts with multiple PKA targets. This suggests that AKAP12 may be crucial in establishing a connection between PKA and its lipogenic substrates. This could explain why, under normal circumstances, perturbations in AKAP12 impact PKA-dependent functions.

RNA sequencing analysis revealed global consequences of *AKAP12* modulation. Inflammation-related signals were major pathways regulated by AKAP12 modulation in the liver and in hepatocytes (Fig. 8). Pro-inflammatory signals upregulated by alcohol in hepatocytes were suppressed by AKAP12 whereas anti-inflammatory signals, IL-10 and LXR/RXR activation pathways, were increased by AKAP12 (Fig. 8b). Overall suppression of MASLD-related networks and inflammatory pathways was observed in the liver of *AKAP12* overexpressing mice with reversed effects observed by PKA-BD CRISPR (Fig. 8e). Our findings on AKAP12 activation as a regulator of liver inflammation are supported by a recent work that showed that hepatocyte-specific deletion of AKAP12 in mice worsened acute liver injury and inflammation brought on by LPS or CCl_4_ exposure.^32^ AKAP12 modulation did not influence xenobiotic stress pathways that were changed by alcohol. CYPs associated with liver injury that are induced in ALD, CYP27A1, CYP4A10, and CYP4A14,^47,48^ did not show significant regulation by AKAP12 (Fig. 8). Even though steatosis and inflammatory signaling are prevented by *AKAP12* overexpression, insignificant response to alcohol dependent CYPs could contribute to the observed lack of normalization of transaminitis.

We investigated the mechanism by which alcohol might destabilize the AKAP12-PKA scaffold. Zhang et al.^38^ recently showed that the PKAR1A regulatory PKA subunit can be phosphorylated at the S77/S83 site by the stress-associated kinase STK25, which may decrease PKA functionality in cardiomyocytes. AKAP12 interacts with PKA regulatory subunits, maintaining PKA activity in hepatocytes, therefore we wondered if STK25 was involved in AKAP12-PKA scaffold modulation. Interestingly, it has been demonstrated that STK25 promotes steatosis in MASLD patients.^49^ Our findings showed that alcohol-enhanced PKAR1A-STK25 interaction and the inhibitory phosphorylation of S77-PKAR1A, which is a gauge of STK25 activity (Fig. 7). Additionally, whereas AKAP12 induction inhibited STK25’s inhibitory PKAR1A-S77 phosphorylation, STK25 depletion recovered the loss of the AKAP12-PKA scaffold following alcohol exposure (Fig. 7). These findings imply that one potential mechanism of AKAP12-PKA scaffold breakdown may be alcohol-mediated STK25 activation.

Our findings summarized in Fig. 8f demonstrate that AKAP12 significantly influences multiple aspects of PKA-dependent lipogenic signaling in the liver. PKA needs AKAP12’s scaffolding activities to preserve lipid homeostasis in the liver in normal and diseased states such as alcohol-associated steatosis. Sustaining the AKAP12-PKA scaffold by designing mimetic peptides for the PKA-BD of AKAP12 could serve as a potential strategy to sustain AKAP12-PKA scaffolding during liver steatosis. A limitation of this study is that even though we have provided evidence of AKAP12’s PKA-BD as a potential regulator of alcohol-associated steatosis, a thorough structure-function investigation of this scaffold would be needed to comprehend the use of AKAP12 PKA-BD as a target for intervention in ALD.

## Materials and methods

### Cell culture and treatments

Cryopreserved, plateable, primary human hepatocytes were purchased from ThermoScientific (Rockford, IL). Cells were passed through percoll-containing density gradient medium (Cytiva, Marlborough) and further washed and resuspended in DMEM + 10%-FBS. Cell suspensions were attached onto PureCol®, Type I Collagen (Advanced biomatrix, Carlsbad CA) at a density of 0.4 million cells per well for 2 h. After 2 h attachment, cells were treated with 100 mM ethanol (Sigma, St. Louis, MO) for 24 h.

### Mouse studies

Standard procedures for the care and use of mice were approved by the Institutional Animal Care and Use Committee at Cedars-Sinai Medical Center (CSMC) (protocol# 8834, PI: Komal Ramani). Female C57BL/6 mice were purchased from Jackson Laboratories. Lieber–DeCarli control (Cat # F1259SP) or ethanol diets (Cat. # F1258SP) were purchased from Bio-Serv, NJ. The protocol for feeding mice a Lieber–DeCarli control diet or an alcohol diet was based on a published model of alcohol feeding^50^ according to the plan in Fig. 5a. During the feeding period, mice were not provided standard chow or water. Four-month-old mice were fed a control diet without alcohol for 4 days to acclimatize the mice to this diet. After 4-day acclimatization period, mice were fed a control diet or a liquid diet containing 5% (vol/vol) ethanol for 10 days. Diets were fed every day during the late afternoon near the dark period of the diurnal cycle to allow the availability of diets during the period of maximal food intake as per published methods.^48^ After 10 days of feeding, mice were orally gavaged with a single binge dose of ethanol (5 g/kg body weight of 20% ethanol). Mice were sacrificed 9 h after gavage. STAM™ mice liver samples from steatosis, MASH, fibrosis, and HCC stages were purchased from SMC Laboratories, Inc. (Tokyo, Japan).

### Human specimens

A human paraffin-embedded tissue array containing 6 normal livers, 19 alcohol, and 5 non-alcohol steatosis livers was purchased from Sekisui Xenotech, Kansas City, MD (TMA.AS Lot No. 1810055).

### Transient overexpression and silencing assays

Human hepatocytes cultured as above were transfected with human CMV-*AKAP12* vector (Vector Builder Inc.) or control mammalian expression vector or EV (Vector Builder Inc.) using lipofectamine™3000 reagent for 48 h according to the manufacturer’s protocol (ThermoScientific). *AKAP12* silencer select siRNA (Cat# 4390824), *STK25* silencer select pooled siRNAs (Cat#4427038, siRNA IDs: s20569, and s20568) or negative control siRNA (Cat# 4390843) (ThermoScientific) were transfected at 12 nM concentration for 48 h using the Lipofectamine™RNAiMAX reagent (ThermoScientific). MirVana™ *miRNA* inhibitors for *MIR-103*, *MIR-186-5p*, *MIR183*, and *MIR1251-5p* (Life Technologies) were transfected at 20 nM concentration for 48 h using RNAiMAX. Ethanol treatment was done during the last 24 h of transfection.

### In vitro *AKAP12* CRISPR gene editing

A synthetic 22-bp small guide RNA (sgRNA) consisting of a crispr RNA (crRNA) and a trans-activating CRISPR RNA (tracrRNA) upstream of a spCas9 PAM was designed using the Edit-R CRISPR system (Horizon Discovery/Dharmacon, Lafayette, CO). A donor RNA for homology-directed repair (PKA-BD HDR) was designed and synthesized using the Edit-R system. Off-target analysis was performed as we previously published.^28^ The sgRNA, donor and a hCMV-SpCas9 vector (Horizon discovery) were co-transfected into cells using the lipofectamine 3000 reagent for 48 h. Cells transfected with spCas9 alone were used as WT controls. Sequences for guide RNAs and donors are provided in Supplementary Table 1.

### In vivo gene editing and *AKAP12* overexpression

Two U6 promoter-sgRNA cassettes upstream and downstream of the DNA region corresponding to AKAP12*-*PKA-BD and a HDR donor containing a deletion of the PKA-BD region, were designed, and cloned by Vector builder Inc., into a control mammalian expression vector (as explained under transient transfection). The same expression vector backbone was used to clone a TBG promoter-saCas9 cassette. Sequences for sgRNA and HDR are provided in Supplementary Table 1. In vivo-jetPEI®-Gal, a galactose-conjugated linear polyethylenimine derivative (Polyplus-transfection S.A, Illkirch, France), was used to conjugate control (EV), human CMV-*AKAP12* or PKA-BD CRISPR/TBG-SaCas9 vectors according to the manufacturer’s protocol. The jetPEI-Gal-DNA complexes were injected intraperitoneally into mice according to the plan in Fig. 5a.

### Actinomycin D chase assay

Control or ethanol-exposed hepatocytes were treated with 5 µg/ml of actinomycin D (Sigma) for 2 h and then cultured for different times, 0 h, 3 h, 6 h, and 20 h in their respective medium. The relative mRNA expression of *AKAP12* or control gene (*HPRT1)* was determined by quantitative real-time RT-PCR as described below.

### Next-generation amplicon sequencing

The efficiency of CRISPR deletion was determined by next-generation amplicon sequencing. Total genomic DNA was subjected to PCR using primers to amplify a 449 bp (human hepatocytes) or a 372 bp (mouse liver) amplicon, including the CRISPR deletion region (Supplementary Table 1). Amplicons from control or CRISPR samples were submitted to Azenta Life Science Inc., CA for next-generation amplicon sequencing as we previously published.^28^ The efficiency of gene editing was calculated as the ratio of WT or CRISPR-edited reads to total reads in the sample. Mismatches were found outside the target amplicon region and were less than 5% of the total reads.

### Quantitative real-time RT-PCR

Total RNA from cells or tissues was converted to cDNA according to our published protocols.^28^ CDNA was subjected to quantitative real-time RT-PCR using pre-designed and validated TaqMan primer probes for human *AKAP12*, mouse *AKAP12*, human *HPRT1,* or mouse *GAPDH*. MicroRNA was purified from cells using the miRNeasy Kit (Qiagen, Valencia CA) according to the manufacturer’s protocol. MicroRNA was converted to cDNA using the TaqMan™ MicroRNA Reverse Transcription Kit with *MIR*-specific primers (Life technologies). *MIR-*specific cDNA was subjected to real-time RT-PCR using validated TaqMan™ MicroRNA Assay primers and probes (Life technologies). The real-time RT-PCR profile for mRNAs and miRs was: initial denaturation: 95 °C for 3 min, 45 cycles: 95 °C, 3 s; 60 °C, 30 s. The relative expression of the target gene versus normalizing gene under control or test conditions was calculated from the cycle threshold (CT) of PCR according to the formula: relative expression = 2^−ΔΔCt^ as we previously published.^28^

### Co-immunoprecipitation and western blotting

Co-immunoprecipitation of AKAP12 and PKA was performed on total protein from cells or tissues using IP lysis buffer (Life Technologies, Cat # 87788). Briefly, 300–500 µg of total protein was pre-cleared using normal rabbit IgG-conjugated protein A/G agarose beads (Santa Cruz Biotechnologies, Santa Cruz, CA) followed by incubation with 2 µg of AKAP12 antibody (proteintech). Cell extracts treated with normal rabbit IgG were used as negative controls. Co-immunoprecipitated proteins and total protein inputs were subjected to western blotting as we published.^28^ Antibodies for western blotting are listed in Supplementary Table 2.

### Crosslinking proteomics

AKAP12 antibody-crosslinked protein A/G beads were used to immunoprecipitate AKAP12 and its interacting proteins from hepatocytes isolated from control or alcohol-fed mouse liver following our previously published protocol.^30^ Immunoprecipitated samples were submitted to Creative Proteomics (Shirley NY) to perform nano LC-MS/MS label-free quantification mass spectrometry using the ultimate 3000 nano UHPLC system coupled with a Q Exactive HF mass spectrometer (ThermoScientific) with an ESI nanospray source. The full scan was performed between 300 and 1650 m/z at the resolution 60,000 at 200 m/z, the automatic gain control target for the full scan was set to 3e6. The MS/MS scan was operated in Top 20 mode using the following settings: resolution 15,000 at 200 m/z; automatic gain control target 1e5; maximum injection time 19 ms; normalized collision energy at 28%; isolation window of 1.4 Th; charge state exclusion: unassigned, 1, >6; dynamic exclusion 30 s. Raw MS files were analyzed and searched against the mouse protein database based on the species of the samples using Maxquant (1.6.2.14). The parameters were set as follows: the protein modifications were carbamidomethylation (C) (fixed), oxidation (M) (variable); the enzyme specificity was set to trypsin; the maximum missed cleavages were set to 2; the precursor ion mass tolerance was set to 10 ppm, and MS/MS tolerance was 0.6 Da.

### Proximity ligation assay (PLA) and immunohistochemistry

Interaction between AKAP12 and PKAR1A or PKAR2A in human tissue arrays was detected by proximity ligation assay. Paraffin-embedded tissue arrays were de-paraffinized and antigen retrieval was performed as we described.^28^ Antibodies were conjugated to PLA plus or minus complementary oligonucleotide arms using the Duolink® In Situ Probemaker MINUS or PLUS kits (Sigma, Cat#DUO92010, or Cat#DUO92009). Mouse AKAP12-minus and rabbit PKAR2A-plus were ligated and detected using Duolink® In Situ Detection Reagents Red (Cat# DUO92008). This was followed by washing and incubation with rabbit AKAP12-plus and mouse PKAR1A-minus and detection with Duolink® In Situ Detection Reagents Green (Cat# DUO92014). Individual PLA interactions in hepatocytes were checked by PLA-probe linked secondary rabbit or mouse IgG as we described.^28^ Tissue slides were processed for immunohistochemistry with AKAP12, albumin, desmin, CD32B, or SaCas9 antibodies followed by detection with mouse or rabbit secondary antibodies, AlexaFluor™488 (green) or AlexaFluor™647 (red) (Supplementary Table 2). Images were visualized using the Keyence BZ-X700 fluorescence Microscope (Keyence Corporation, IL).

### PKA activity assay

PKA kinase activity in extracts of cultured hepatocytes or liver tissue was detected by a phospho-peptide substrate ELISA kit using a phospho-specific antibody and HRP-conjugated secondary according to the manufacturer’s protocol (Abcam, Cat#: ab139435). The assay was developed with TMB substrate, and the color intensity was measured in a microplate reader at absorbance, A450 nm. The relative kinase activity was calculated by the formula: *(Average absorbance of sample duplicates-Average absorbance of blank duplicates)/Amount of crude protein used per assay*.

### Nile red staining

Intracellular lipids in attached hepatocytes under different treatment conditions were stained using Nile red stain according to the manufacturer’s protocol (Abcam).

### Triglyceride content

Triglyceride content was measured using the Triglyceride-Glo™ Assay kit (Promega, Madison WI). The reaction measured glycerol released by enzymatic action of lipase on triglycerides. Gycerol release was coupled to a glycerol kinase/dehydrogenase reaction that produces NADH which facilitated a reductase dependent reaction converting reductase substrate-bound pro-luciferin to luciferin. Luciferin was detected in a luciferase reaction using Ultra-Glo™ Luciferase and ATP in a plate luminometer. The nmole of triglyceride normalized to total protein content was calculated from the luminescent signal of standard triglyceride of known concentration.

### P-CREB DNA binding activity assay

Nuclear extracts from liver tissues were prepared by the nuclear extraction kit (Cayman Chemical, Cat #10009277). DNA binding activity of P-CREB-S133 was examined in nuclear extracts using the CREB (P-133) transcription factor assay kit (Cayman, Cat# 10009846). Briefly, a 96-well plate coated with a double-stranded CRE DNA element was treated with nuclear extracts followed by incubation with P-CREB-S133 antibody and development with HRP-based secondary. The assay was quantified using a plate reader at an absorbance of 450 nm and the absorbance units per mg of nuclear protein were calculated.

### CYP2E1 activity assay

CYP2E1 activity was measured in liver microsome fractions as described.^51^ Briefly, 1 mg of microsomal protein was incubated in a 100 ul reaction containing 100 mM phosphate buffer and 0.2 mM Para nitrophenol. The reaction was initiated at 37 °C by addition of 1 mM NADPH and terminated after 20 min by addition of trichloroacetic acid to a final concentration of 1%. The reaction was centrifuged at 5000 *g* for 10 min to remove precipitated protein and supernatant was treated with 10ul NaOH. The absorbance of the pink-yellow p-nitrocatechol product formed was measured spectrophotometrically at 510 nM. The concentration of p-nitrocatechol was determined from the extinction coefficient 9.53 mM^−1^ cm^−1^. The nmol per mg protein was calculated from the formula: *Absorbance at 510* nm/(Extinction coefficient) * (Dilution factor) * (10^6/Molecular weight of CYP2E1).

### Cell-based ELISA assay

Cell-based ELISA assay reagents were purchased from Sigma and a protocol to measure phospho-S77-PKAR1A modification in hepatocytes was followed according to the manufacturer’s instructions. Briefly, human hepatocytes cultured in 96-well plates were fixed, permeabilized, and endogenous peroxidase activity was quenched using a quenching buffer (Sigma) followed treatment with a blocking buffer. A group of cells was not treated with antibodies and was used to estimate the total protein level. The other groups were incubated with phospho-S77-PKAR1A, PKAR1A antibody followed by HRP-conjugated secondary. Samples were developed with TMB substrate. The ELISA assay was quantified using a plate reader at an absorbance of 450 nm and the absorbance units per µg protein were calculated.

### Histopathological examination

Formalin-fixed liver tissues were paraffin-embedded and sections were stained with hematoxylin and eosin (H&E) by the Cedars-Sinai Biobank and Research Pathology Resource. Perilipin-2 lipid droplet marker staining was performed by immunohistochemistry using perilipin-2 or PLIN2 antibody followed by detection using the mouse and rabbit-specific HRP/DAB detection IHC Kit (Abcam, Cat# ab64264). Sections were subjected to BODIPY™ as per instructions from the manufacturer (ThermoScientific).

### ALT/AST level

Plasma ALT and AST levels were measured by using colorimetric assay kits from Cayman Chemicals, MI as we published previously.^28^

### RNA sequencing

Total RNA was isolated from mouse hepatocytes or total liver and processed for RNA sequencing by Azenta Life Science Inc. MRNA enriched via poly(A) selection were fragmented and subjected to random priming followed by first and second-strand cDNA synthesis. Samples were then end-repaired, 5’-phosphorylated, and dA-tailed and ligated with adapters. RNA sequencing libraries were prepared on an Illumina 2x150bp configuration. Trimmed reads were mapped to the mouse genome and hit counts for genes/exons were determined. Gene hit counts were compared between different samples to define differentially expressed genes (DEGs). The log_2_-fold change (log_2_FC) is: Log_2_(Test mean normalized counts/Control mean normalized counts).

### Statistical analysis

Western blots were quantified by densitometry ImageJ software (NIH). PLA staining was analyzed in a blinded manner by two individuals and quantified using ImageJ according to our published protocols.^28^ Graphs showing individual experiments and their mean±S.E were plotted using GraphPad Prism 10.2.3, GraphPad software. Statistical analysis was performed using two-tailed Student’s *t*-test for single pair comparisons and two-way or 3-way ANOVA with Tukey’s post hoc analysis for comparison between multiple groups. Significance was defined as *P* < 0.05. Individual data points and statistical calculations are presented in Data S4.

## Supplementary information

Supplementary materials

Data S1

Data S2

Data S3

Data S4

## Data Availability

We declare that data supporting the findings of this study are available within this manuscript and its supplementary information files. The RNA sequencing data (Figure 8, Data S2 and 3) discussed in this publication have been deposited in NCBI’s Gene Expression Omnibus^52^ and are accessible through GEO Series accession number GSE290266 (https://www.ncbi.nlm.nih.gov/geo/query/acc.cgi?acc=GSE290266).
