# Supplementary material for: The AKAP12-PKA axis regulates lipid homeostasis during alcohol-associated liver disease
Source: Signal Transduct Target Ther. 2025 Apr 9;10:109. doi: 10.1038/s41392-025-02202-1 (PMC11979000; doi:10.1038/s41392-025-02202-1)
Supplement: Supplementary file 1 — Supplementary materials [file 41392_2025_2202_MOESM1_ESM.docx]

Supplementary Materials for

**The AKAP12-PKA axis regulates lipid homeostasis during alcohol-associated liver disease**

Chandana Thimme Gowda, Mallikarjuna Siraganahalli, Jiaohong Wang, Youngyi Lim, Maria Lauda Tomasi, Nirmala Mavila, Komal Ramani

Correspondence to: [komal.ramani@cshs.org](mailto:komal.ramani@cshs.org)

**This PDF file includes:**

Figures. S1 to S21

Tables S1 and S2

Captions for Data S1 to S3

**Other Supplementary Materials for this manuscript include the following:**

Data S1: Differentially expressed protein (DEP) analysis

Data S2: Differentially expressed genes sequencing analysis of mouse hepatocytes (DEGseq)

Data S3: Differentially expressed genes sequencing analysis of mouse liver (DEGseq).

Data S4: Quantitative raw data for graphs and anova/post-hoc statistical analysis for all figures


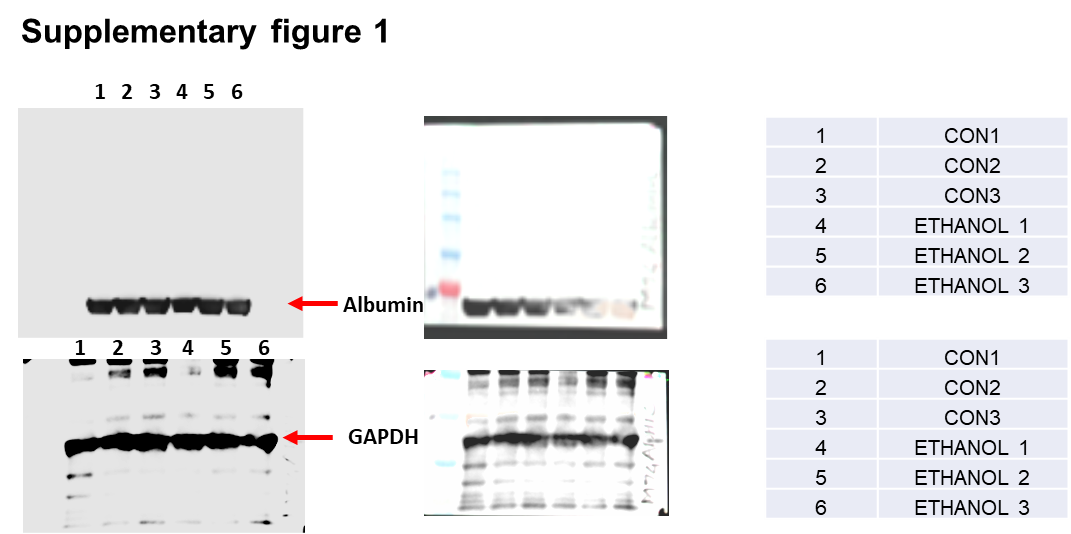


**Supplementary figure 1. Expression of albumin in control or ethanol-treated hepatocytes.** Human hepatocytes were treated with alcohol as in methods and total protein was subjected to western blotting for albumin or GAPDH using specific antibodies. Images are raw uncropped blots (left) and blots merged with marker (right).


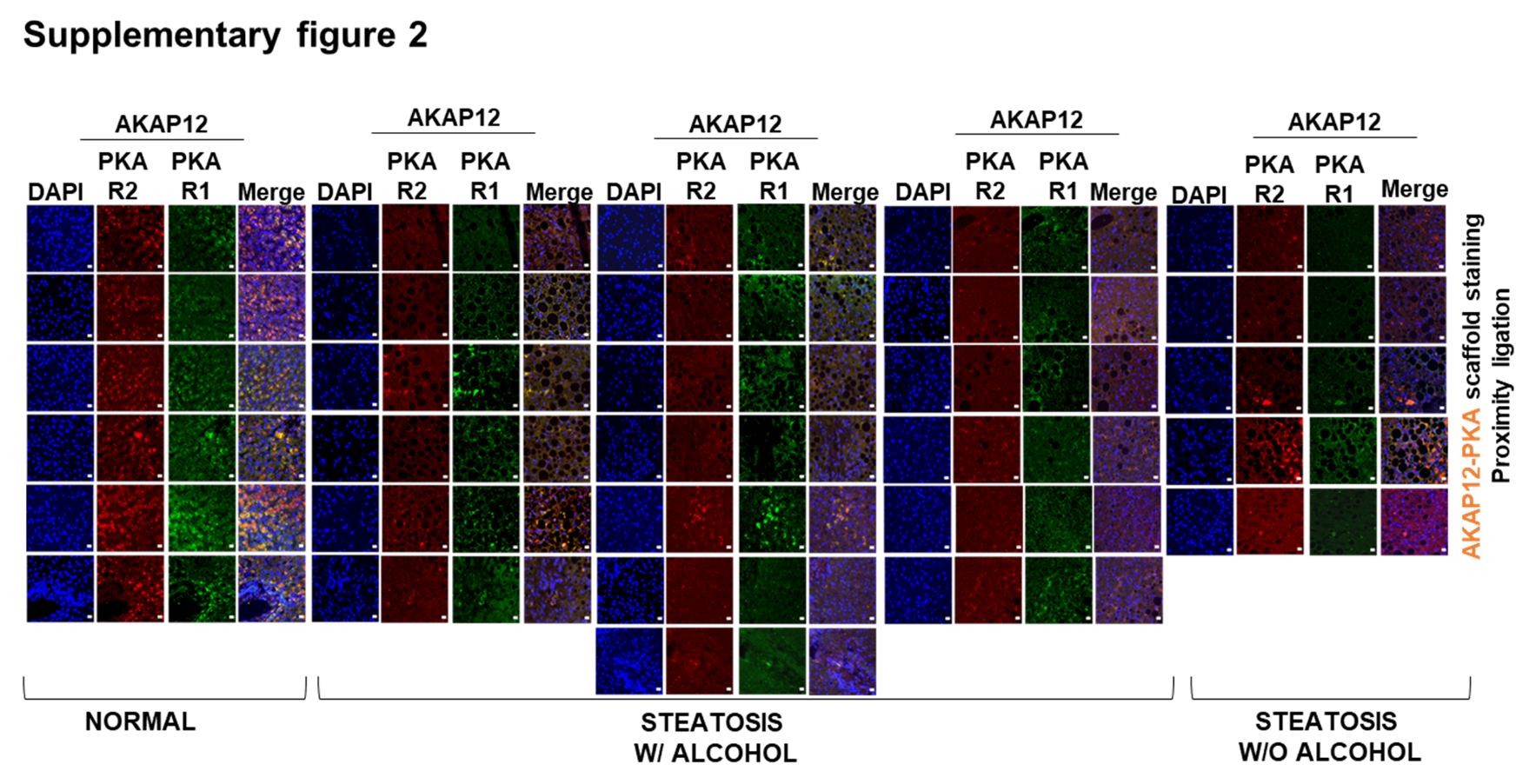


**Supplementary figure 2. AKAP12-PKA scaffold PLA staining in human steatosis W/ or W/O alcohol use and normal tissues.** Human liver steatosis array containing 6 normals, 19 steatosis W/ alcohol and 5 steatosis W/O alcohol tissues was stained with PLA probes as explained in figure 1c legend. The entire tissue array staining and imaging at 40X magnification is shown, scale bar: 10µm.

**
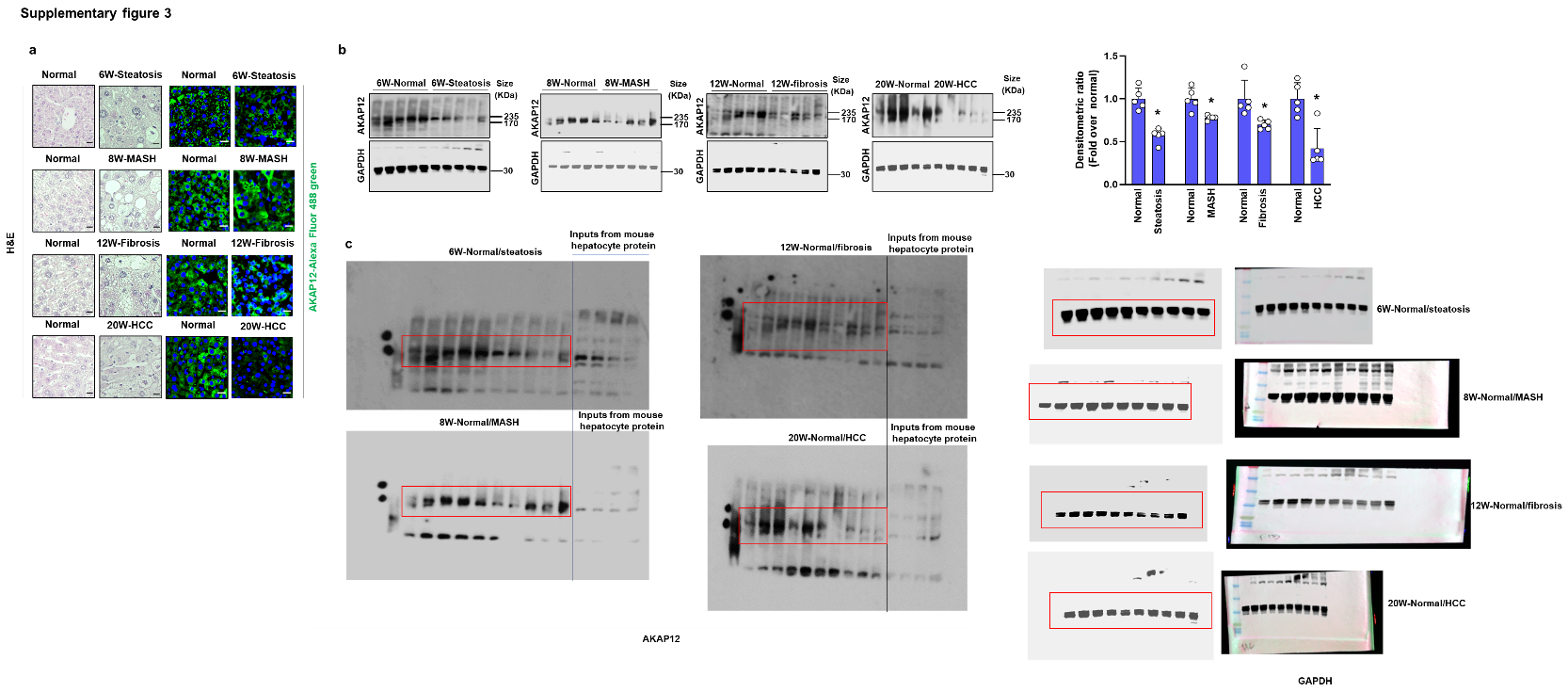
**

**Supplementary figure 3. AKAP12 expression in STAM™ model mouse liver. a.** Tissue slides from STAM™ livers at steatosis (6-weeks, 6W), MASH (8W), fibrosis (12W) and HCC (20W) were stained with H&E or with AKAP12 antibody and AlexaFluor™ 488 secondary as described under methods. Images are representative of 5 livers per group. **b**. Total protein from the tissues in ‘a’ above were subjected to western blotting and densitometric quantification using ImageJ. *p<0.001 vs. respective normal controls. **c**. Raw uncropped images of blots in ‘B’ above are shown with the bands highlighted in red box.


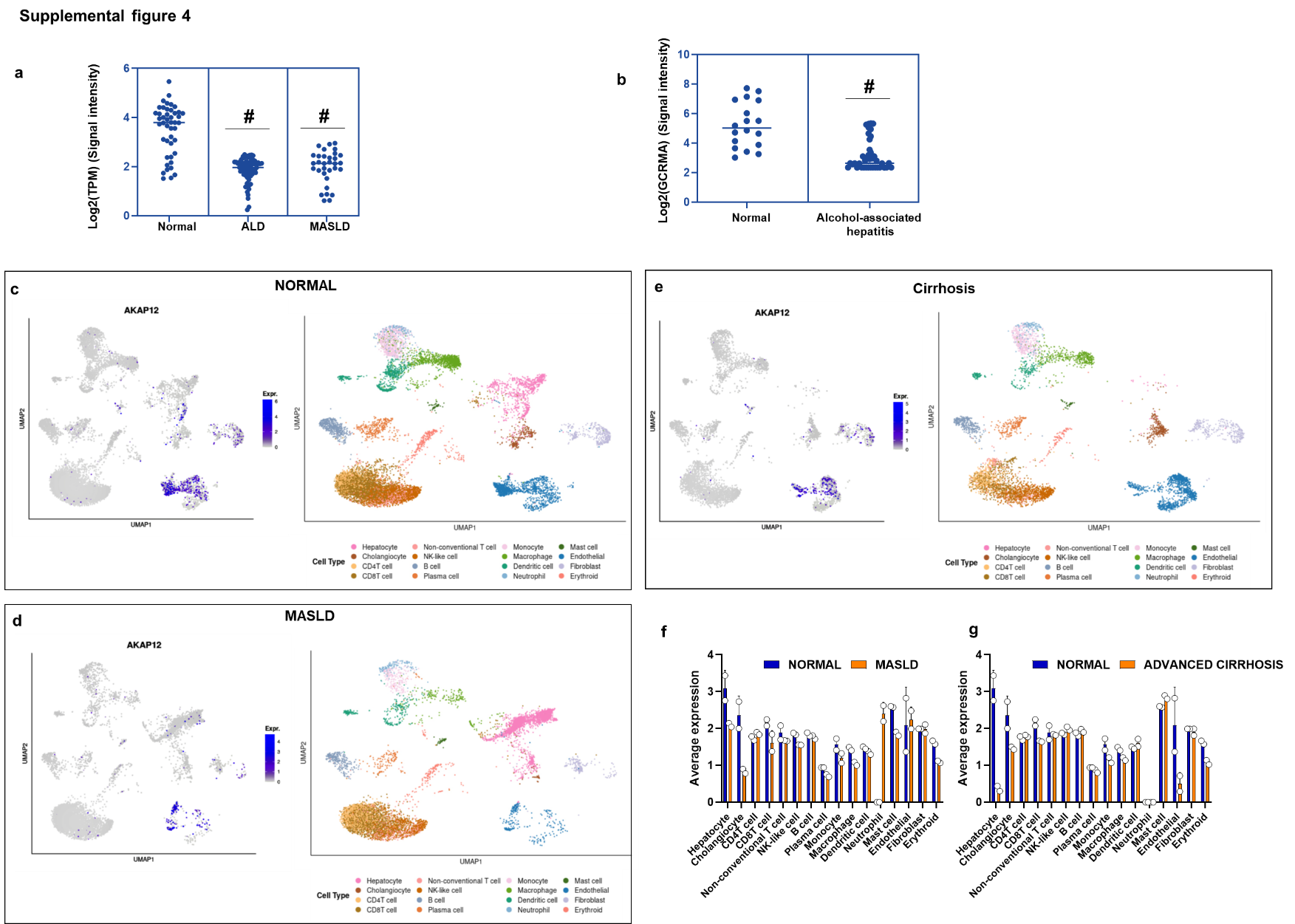


**Supplementary figure 4. Transcriptomic and RNA sequencing analysis of *AKAP12* from publicly available datasets of human liver disease. a.** Bulk RNA sequencing data of *AKAP12* from GEPliver database as described in results from normal, ALD and MASLD human samples. The data is signal intensity expressed as log_2_ (TPM) where TPM=transcripts per million. **b**. Transcriptomic analysis of *AKAP12* from the GEO dataset, GDS4389. The signal intensity is expressed as log_2_(GCRMA) where GCRMA= GeneChip Robust Multi-array Analysis with GC content correction. #p<0.001 vs. normal. **c, d, e**. Single cell RNA sequencing analysis of *AKAP12* from GEPliver as described in results. C=normal, D=MASH, E=Cirrhosis. **f, g**. Average expression of *AKAP12* in cell types from ‘c, d, e’ from two datasets.


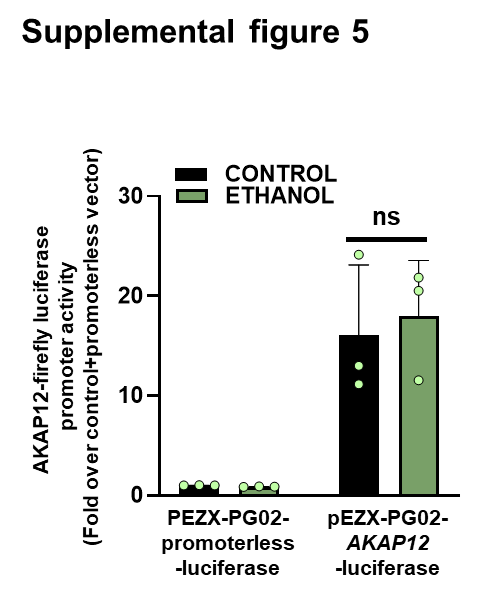


**Supplementary figure 5. AKAP12 promoter activity in control or alcohol-treated hepatocytes.** Human hepatocytes were transfected with a negative control (promoterless) or *AKAP12-*luciferase vector and treated with ethanol as described under methods. Data is representative of 3 independent experiments.


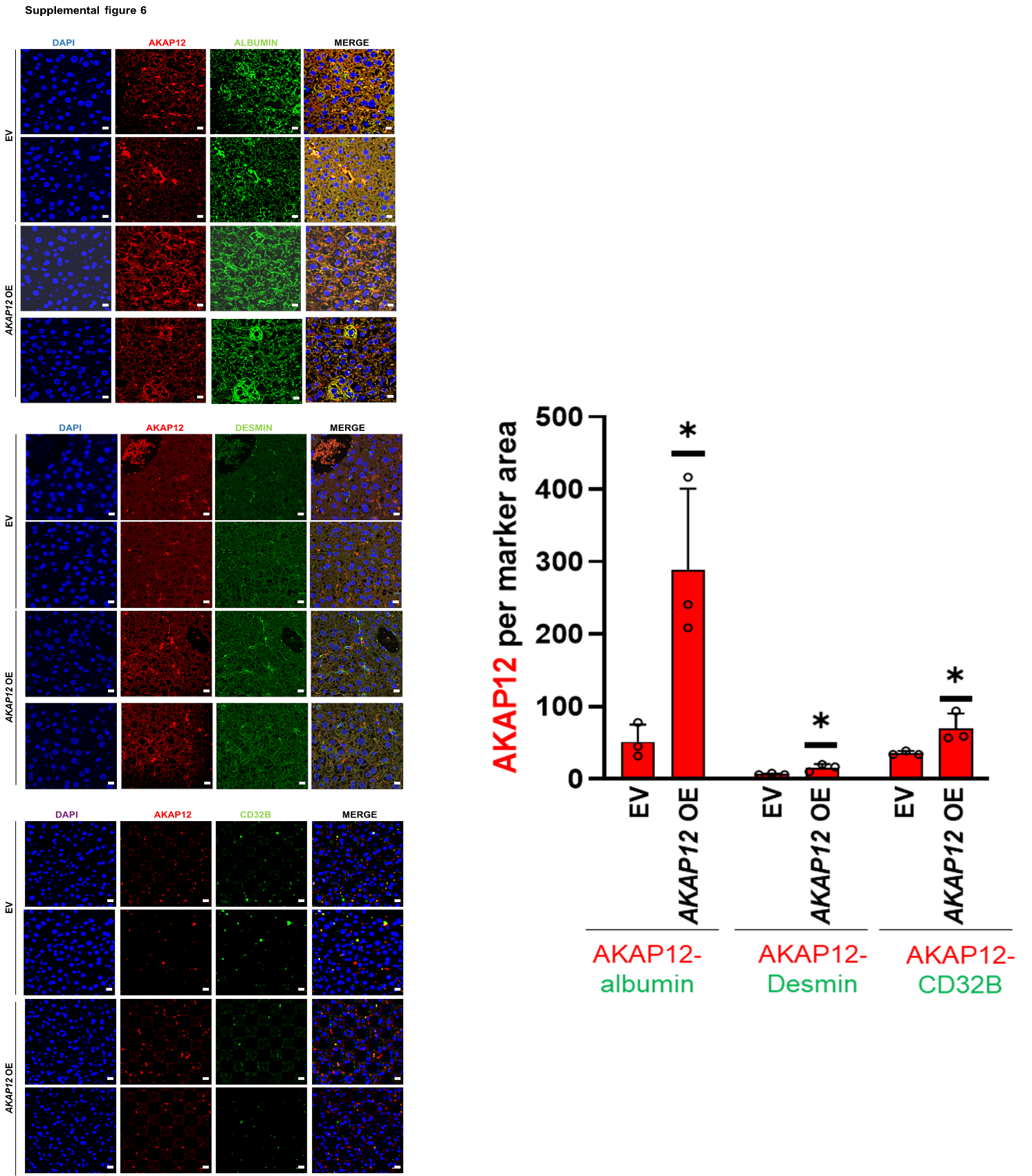


**Supplementary figure 6. Co-expression of AKAP12 with albumin, desmin or CD32B markers in mouse liver.** Liver tissues were costained with AKAP12 and hepatocyte marker, albumin, HSC marker, desmin or liver endothelial cell marker, CD32B as in methods. The tissue staining and imaging at 200X magnification is shown, scale bar: 15µm. The co-localized areas were quantified using ImageJ. Data is representative of 6 experiments. *p<0.05 vs. EV.


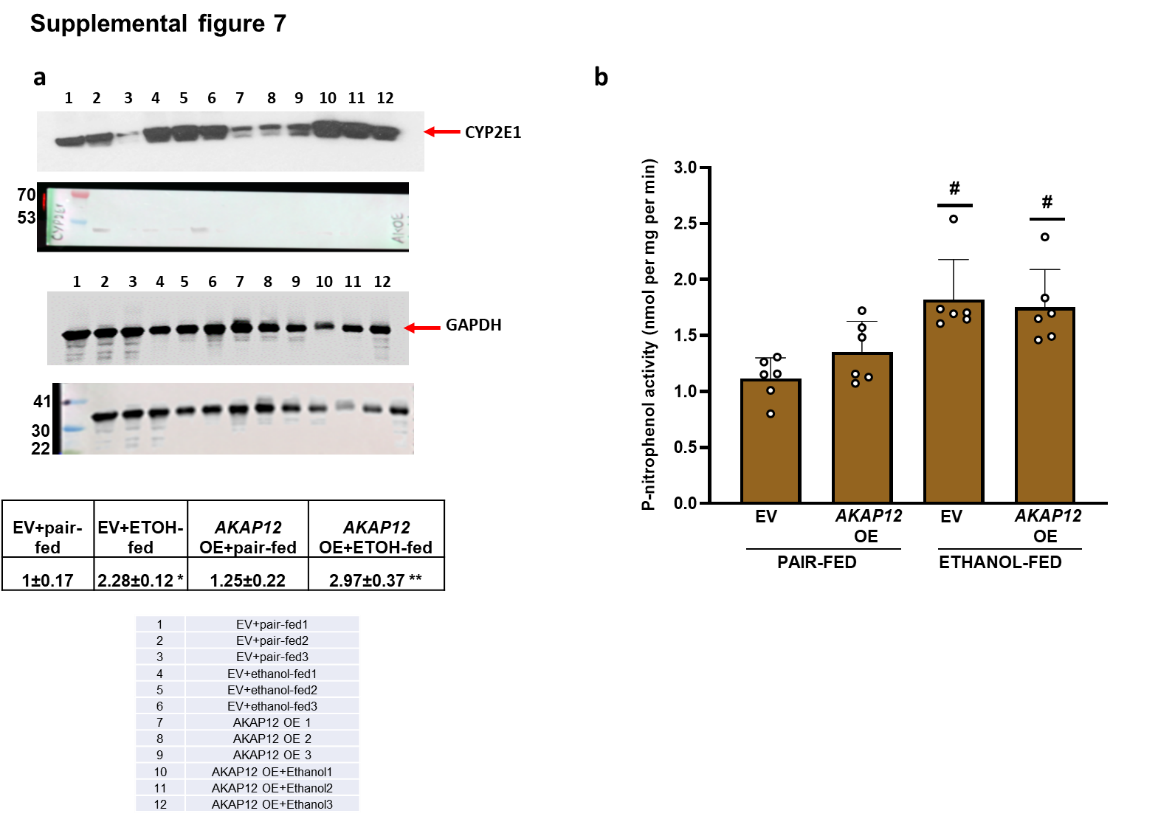


**Supplementary figure 7. CYP2E1 expression and activity in *AKAP12-*overexpression livers.** Mice were injected with *AKAP12* vector as described in methods. **a**. CYP2E1 protein expression in liver protein was checked by western blotting from 6 experiments. **p<0.001, *p<0.05 vs. EV+pair-fed. **b**. CYP2E1 activity in liver extracts was measured as described in methods. Data is representative of 6 experiments. #p<0.01 vs. EV+pair-fed.


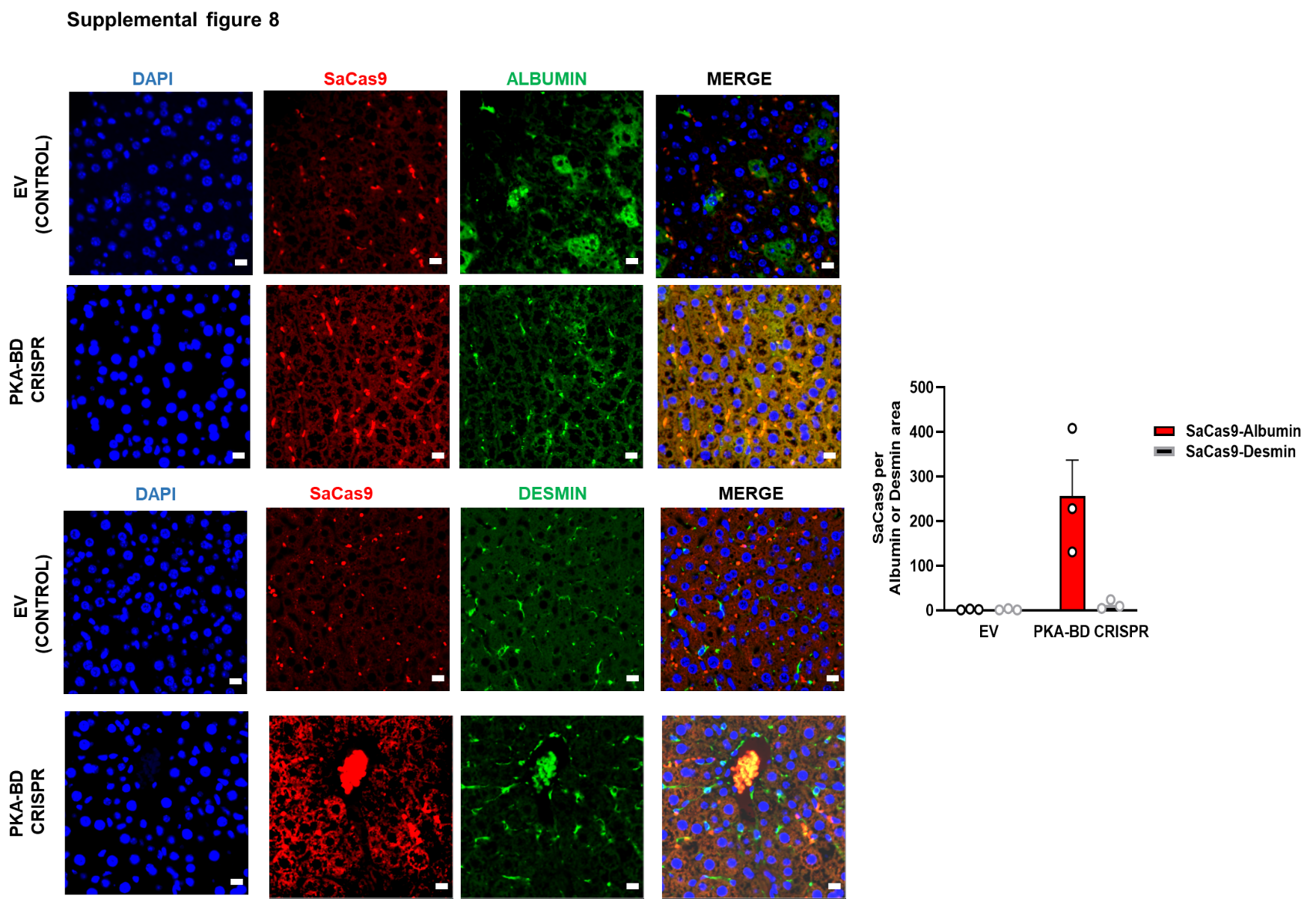


**Supplementary figure 8. Co-expression of SaCAS9 with albumin or desmin markers in mouse liver.** Liver tissues were co-stained with SaCas9 and hepatocyte marker, albumin or HSC marker, desmin as in methods. The tissue staining and imaging at 200X magnification is shown, scale bar: 15µm. The co-localized areas were quantified using ImageJ.


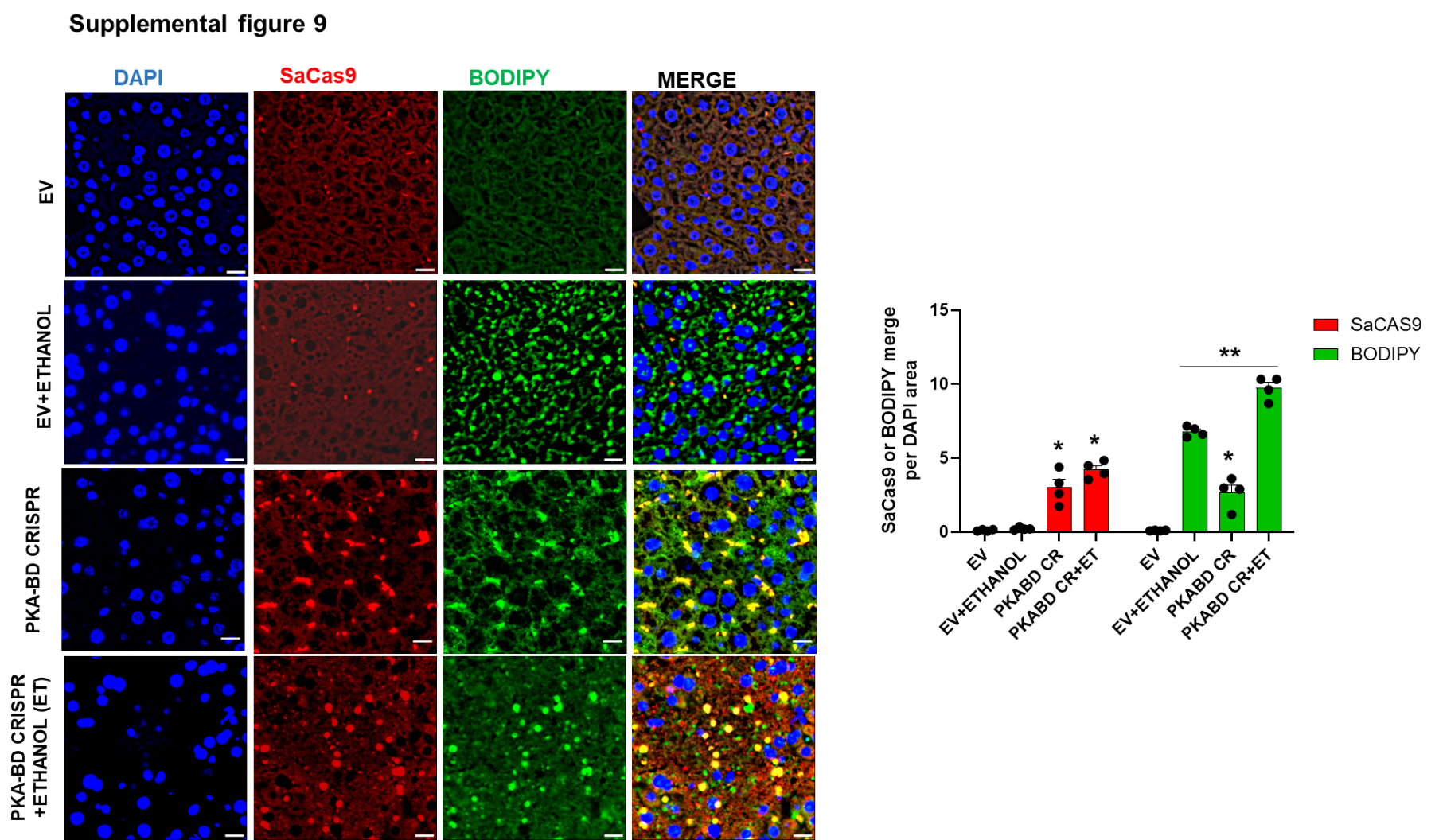


**Supplementary figure 9. Co-staining of PKA-BD CRISPR enzyme, SaCas9 with lipids in mouse liver.** EV, EV+ethanol, PKA-BD CRISPR or PKA-BD CRISPR+ethanol treated livers were co-stained with SaCas9 antibody (alexa Fluor 688 red) and BODIPY lipid stain as described under methods. The tissue staining and imaging at 200X magnification is shown, scale bar: 25µm. Data are representative of 4 experiments, *p<0.001 vs. EV (SaCas9), **p<0.001 vs. EV (BODIPY).


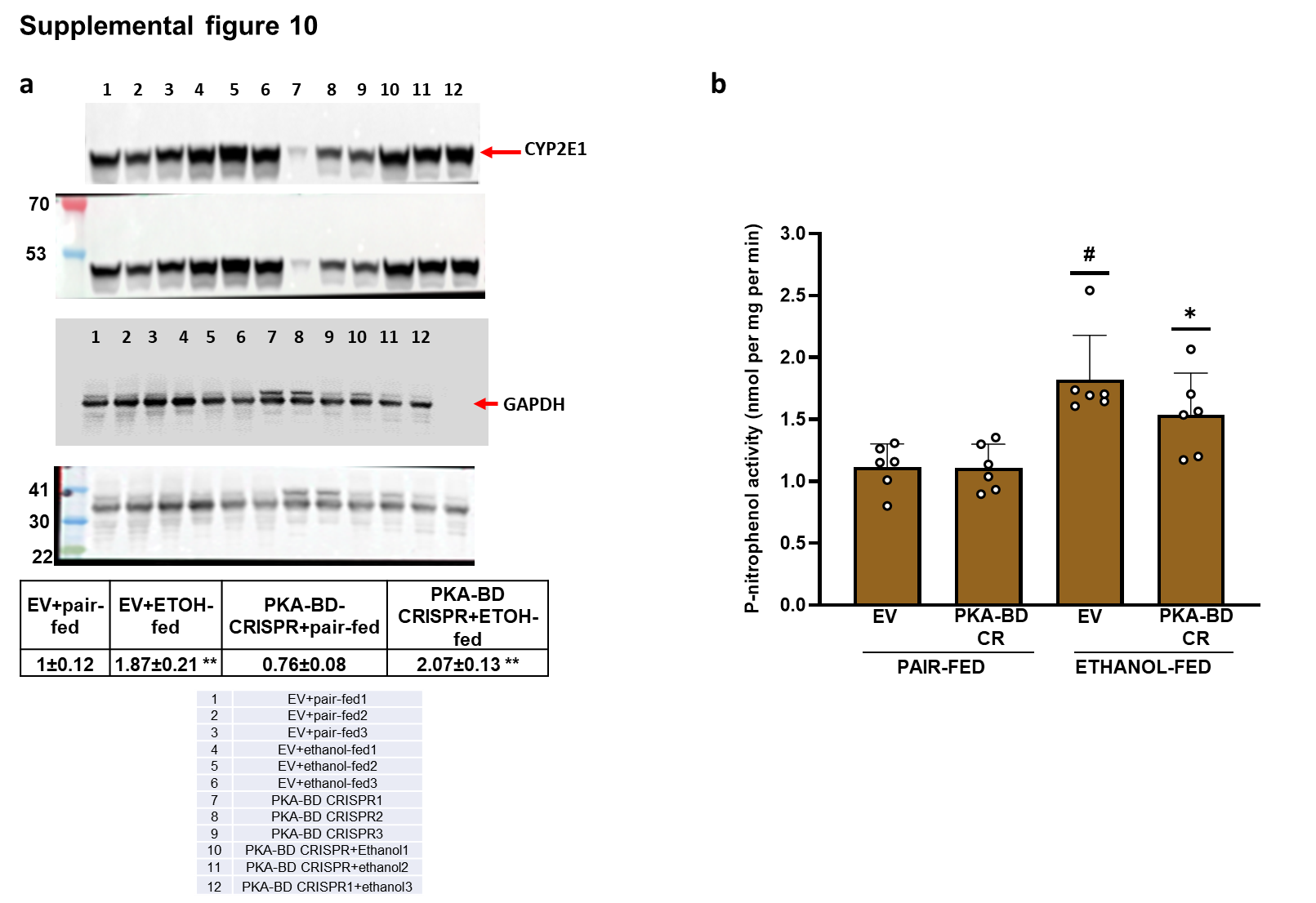


**Supplementary figure 10. CYP2E1 expression and activity in PKA-BD CRISPR livers.** Mice were injected with PKA-BD-CRISPR livers as described in methods. **a**. CYP2E1 protein expression in liver protein was checked by western blotting from 6 experiments. **p<0.001, *p<0.05 vs. EV+pair-fed. **b**. CYP2E1 activity in liver extracts was measured as described in methods. Data is representative of 6 experiments. #p<0.01, *p<0.05 vs. EV+pair-fed.


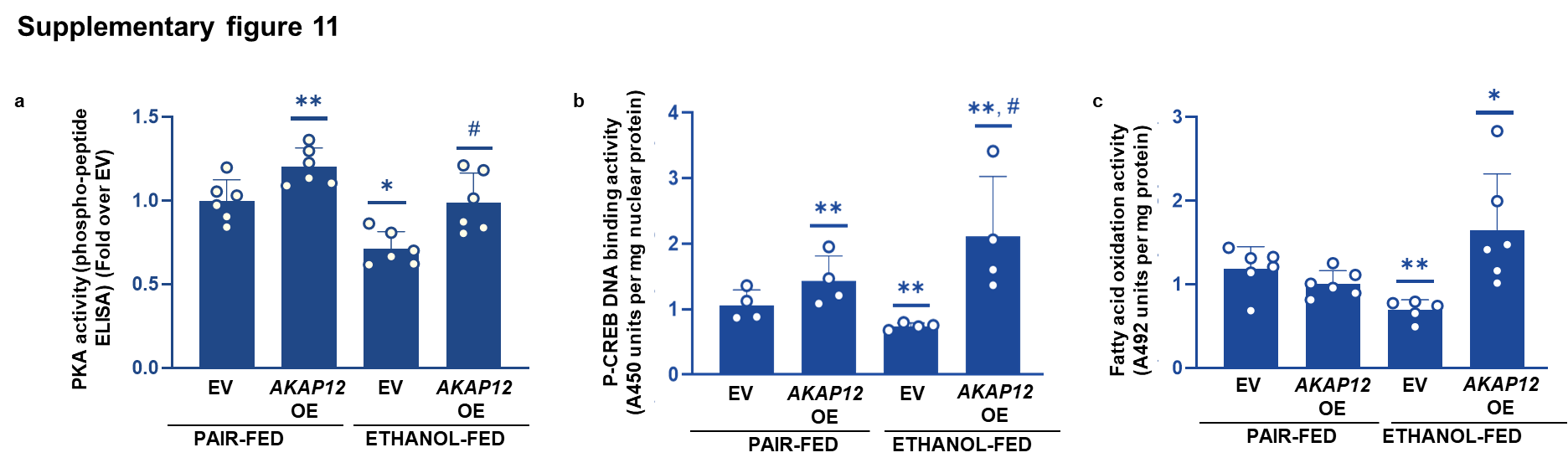


**Supplementary figure 11. Forced expression of *AKAP12* alters PKAs lipogenic targets under basal and ALD conditions.** Mice were injected with *AKAP12* vector as described in Figure 5a legend. **a**. Liver extracts were assayed for PKA activity using a phospho-peptide ELISA as described under methods. Data represents the PKA activity as fold over EV from 6 experiments **p<0.05 vs. pair fed, *p<0.01 vs. pair-fed, #p<0.01 vs. ethanol fed. **b**. Nuclear extracts prepared from liver were processed to measure P-CREB DNA binding activity as described under methods. Data are represented as the A450 absorbance units per mg of nuclear protein from 4 experiments. **p<0.05 vs. EV+pair-fed, #p<0.05 vs. EV+ethanol-fed, **c**. FAO activity in total extracts was measured as described in methods. The The data represents FAO activity (A492 absorbance units per mg liver protein) from 5 to 6 experiments. **p<0.01 vs. pair fed, *p<0.05 vs. ethanol.


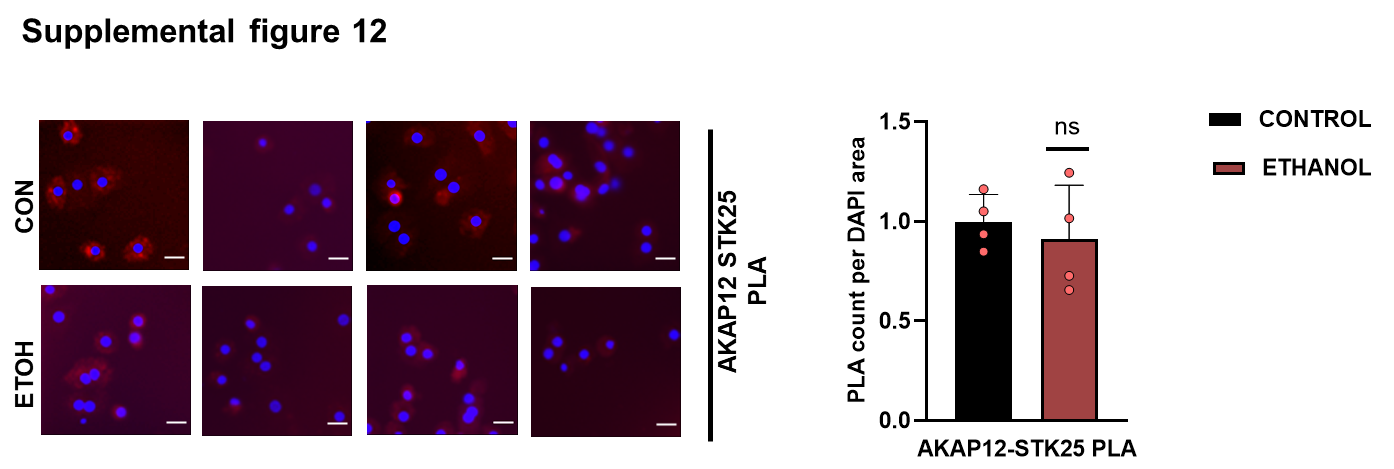


**Supplementary figure 12. AKAP12-STK25 scaffold is unchanged upon alcohol exposure.**

Hepatocytes under control or ethanol conditions were subjected to PLA staining to detect interaction between AKAP12 and STK25 as described in methods. PLA staining of AKAP12-STK25 interaction (red) and nuclear DAPI (blue) was quantified by ImageJ and represented as the PLA count per DAPI area from 4 experiments.


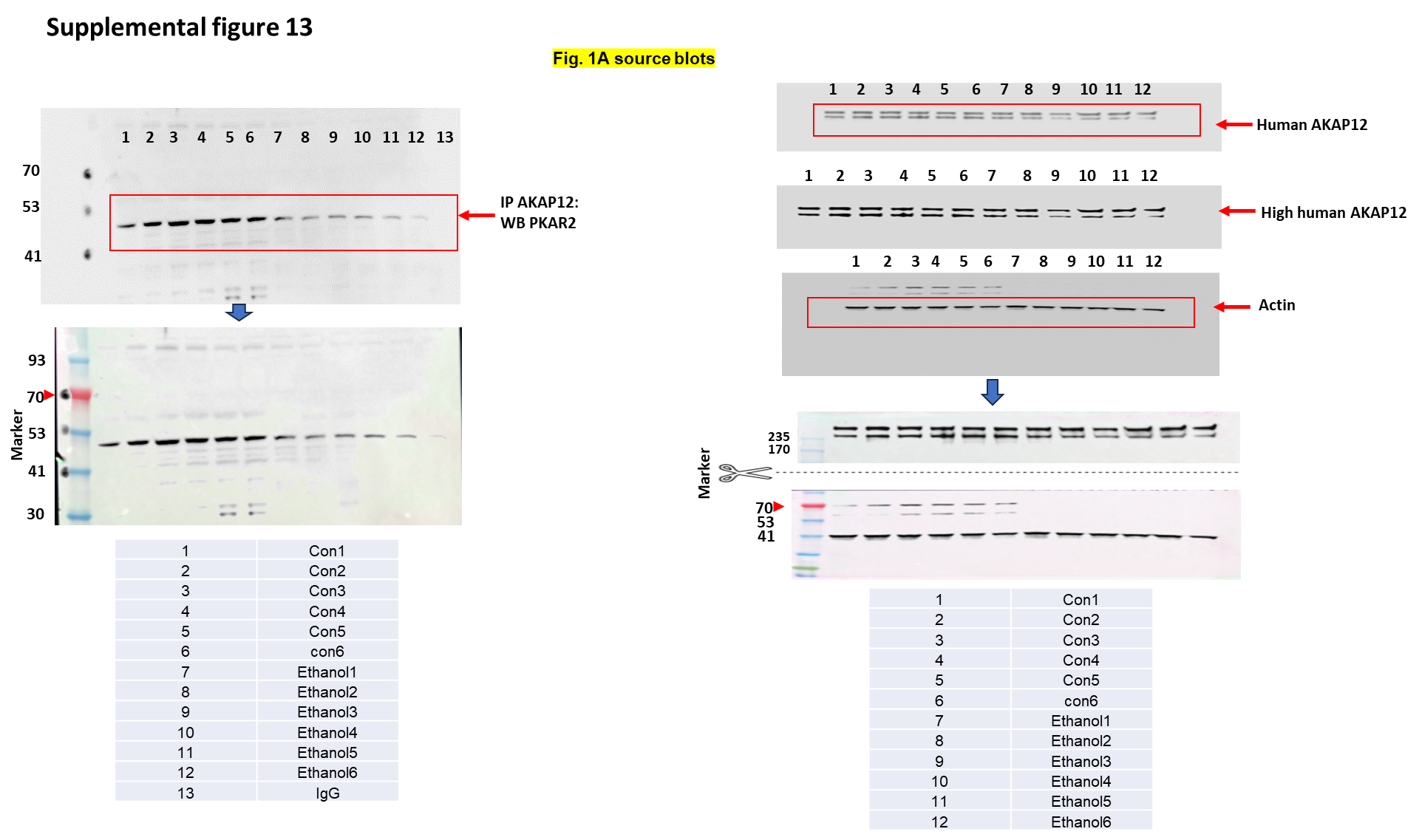


**Supplementary figure 13**. Uncropped blots for figure 1A with markers.


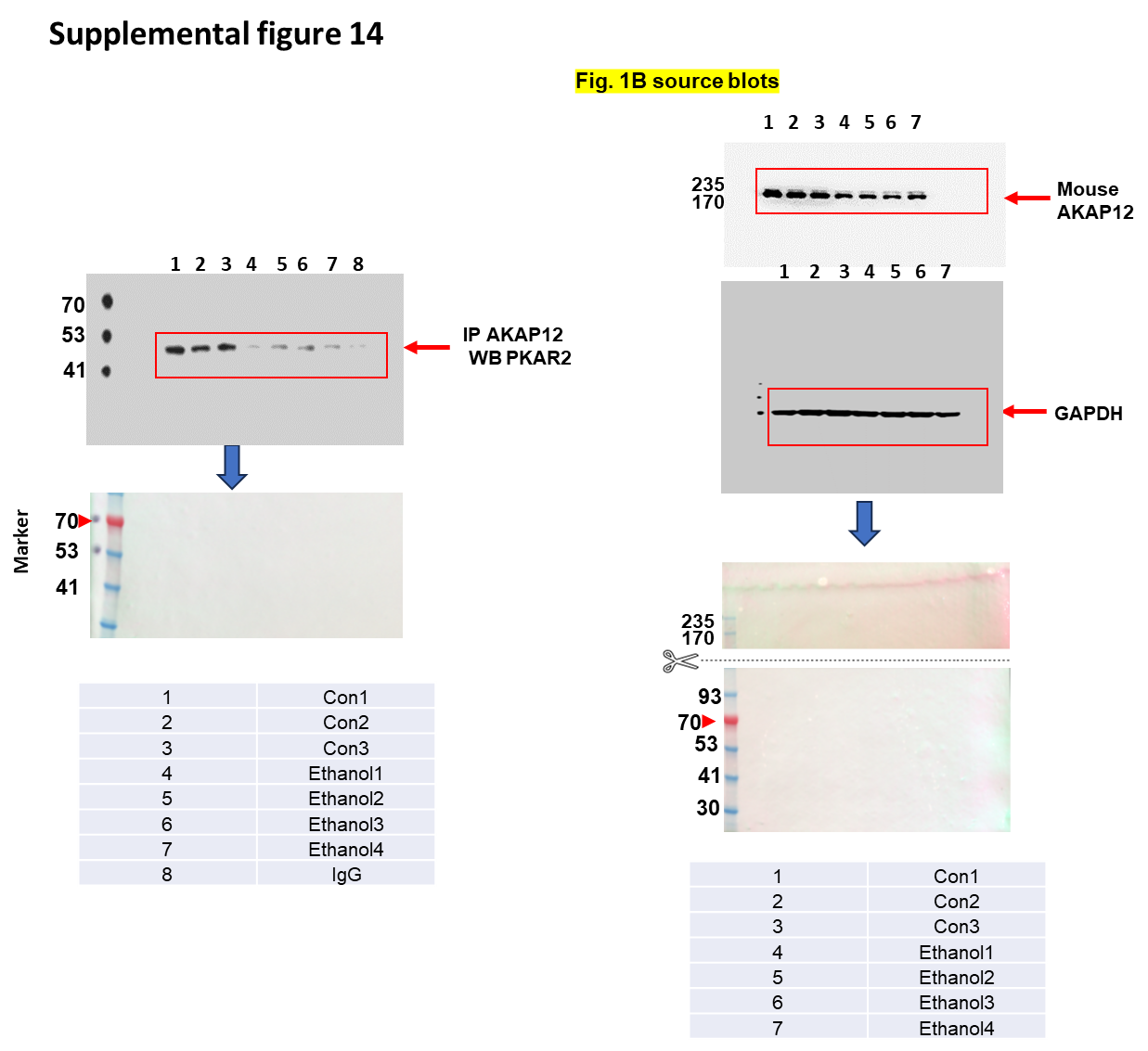


**Supplementary figure 14**. Uncropped blots for figure 1B with markers.


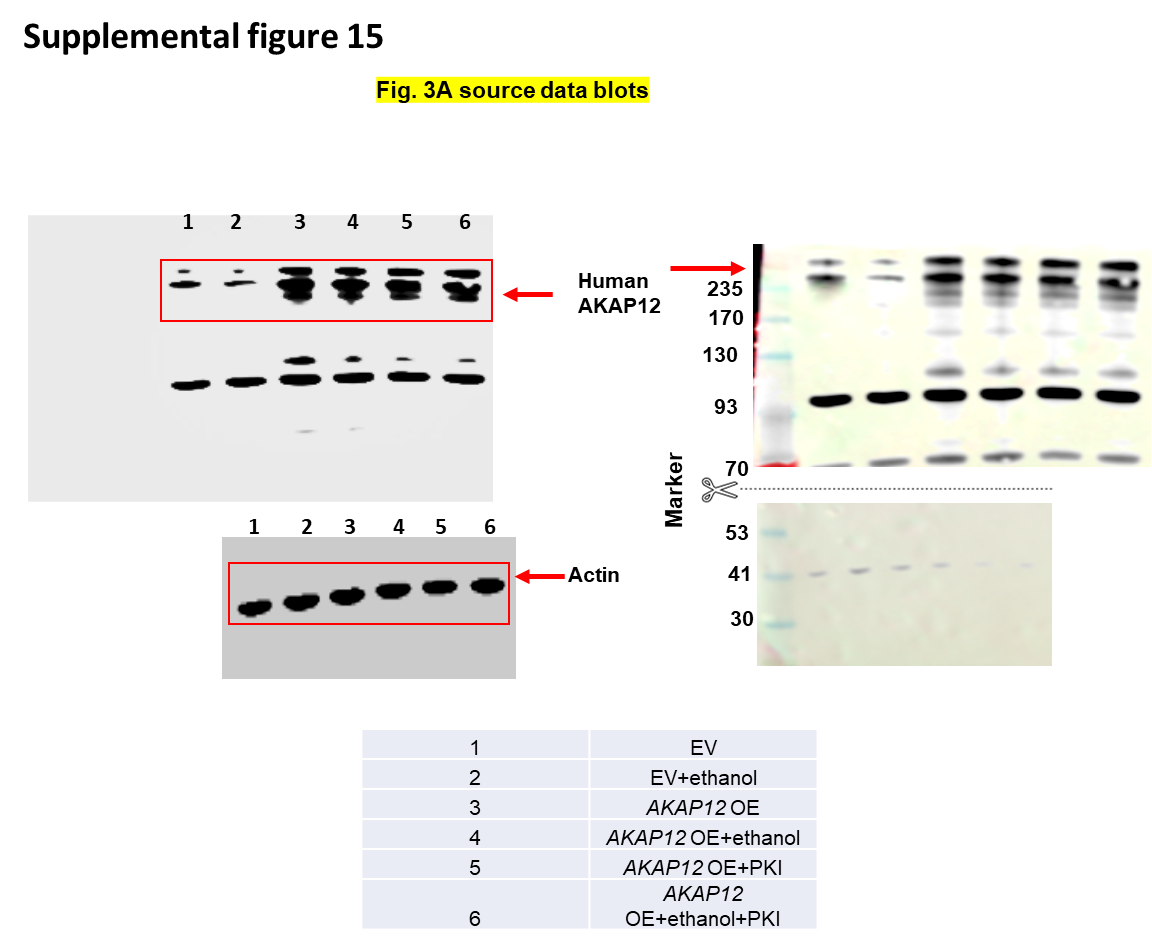


**Supplementary figure 15**. Uncropped blots for figure 3A with markers.


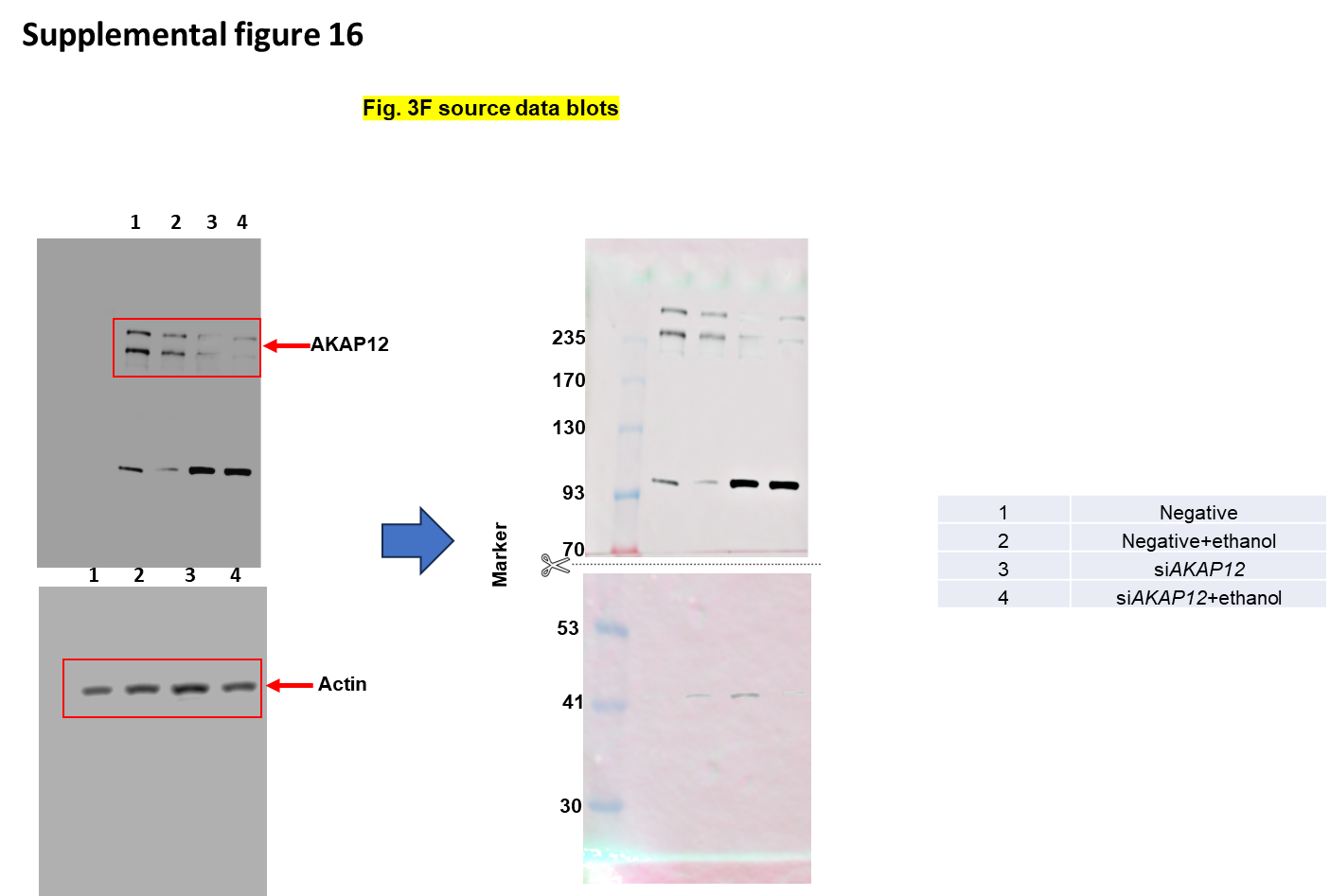


**Supplementary figure 16**. Uncropped blots for figure 3F with markers.


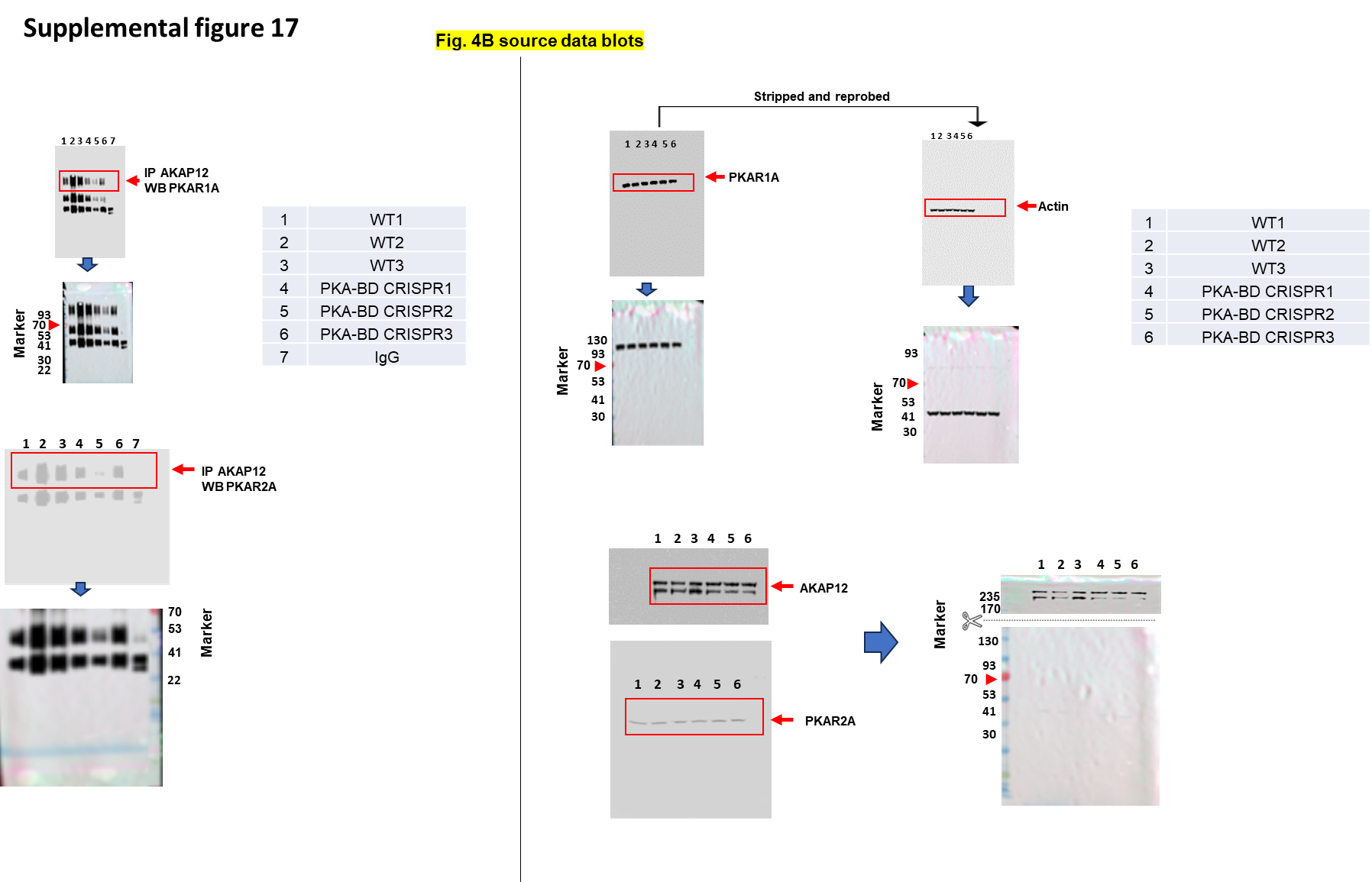


**Supplementary figure 17**. Uncropped blots for figure 4B with markers.


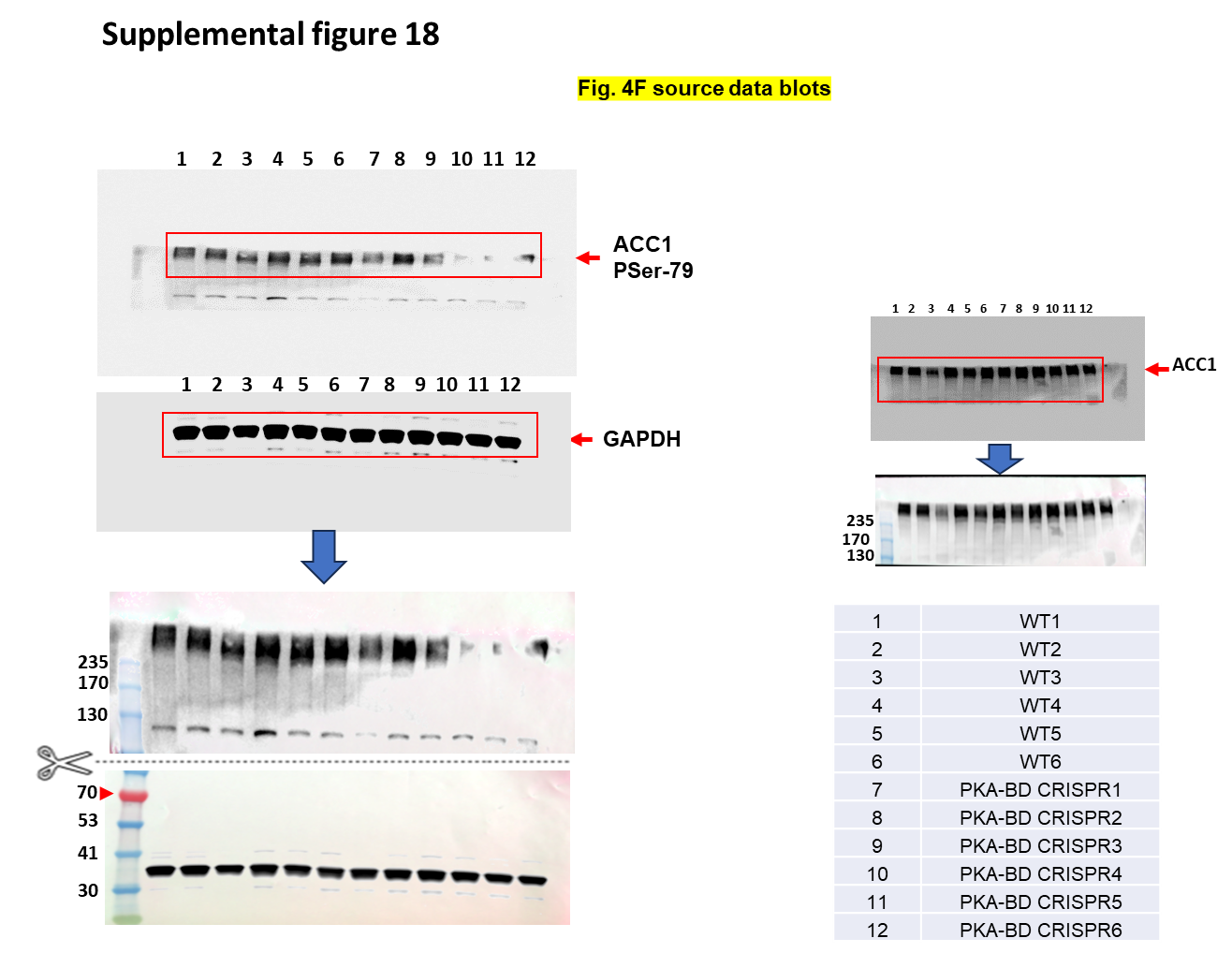


**Supplementary figure 18**. Uncropped blots for figure 4F with markers.


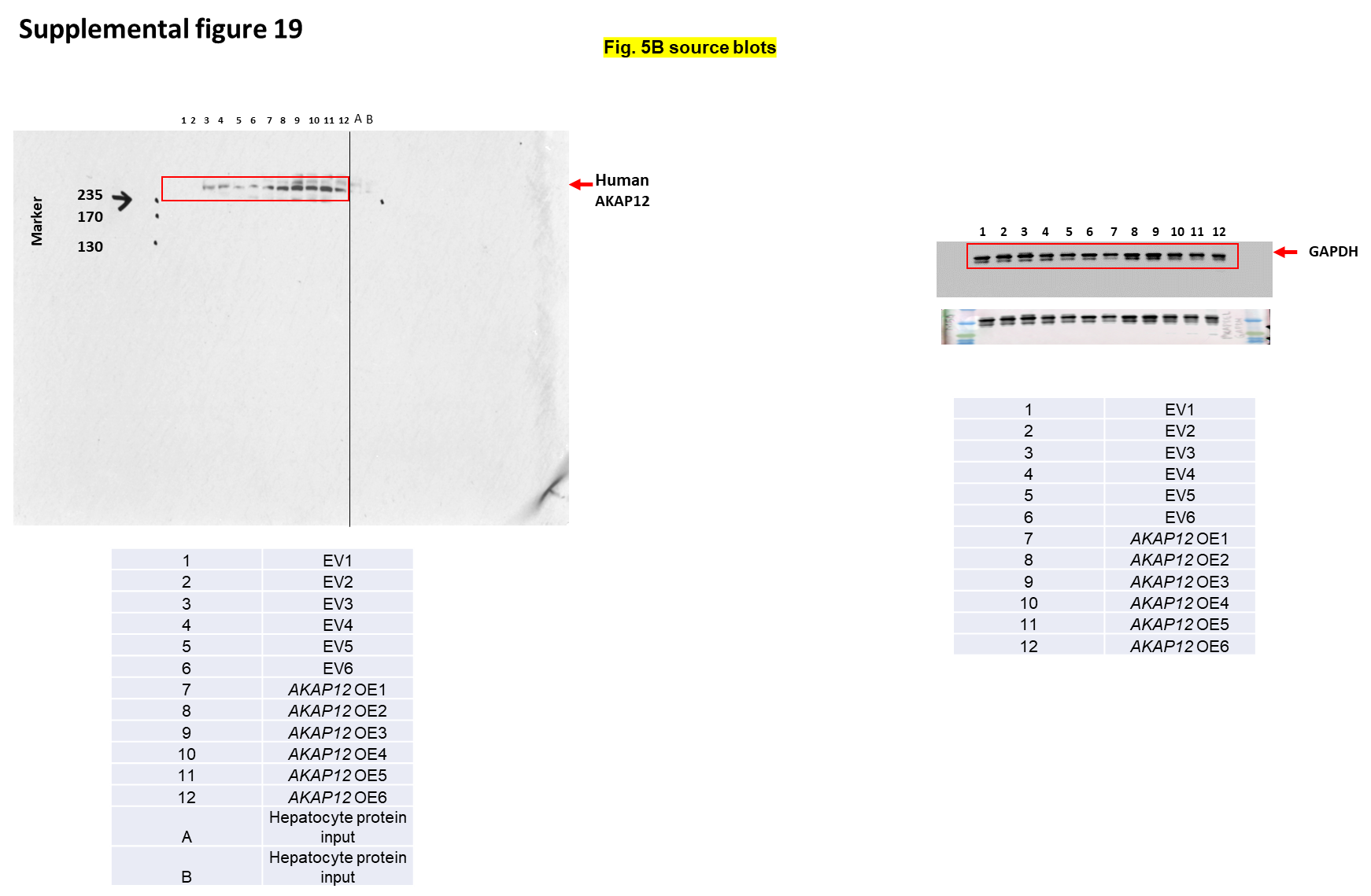


**Supplementary figure 19**. Uncropped blots for figure 5B with markers.


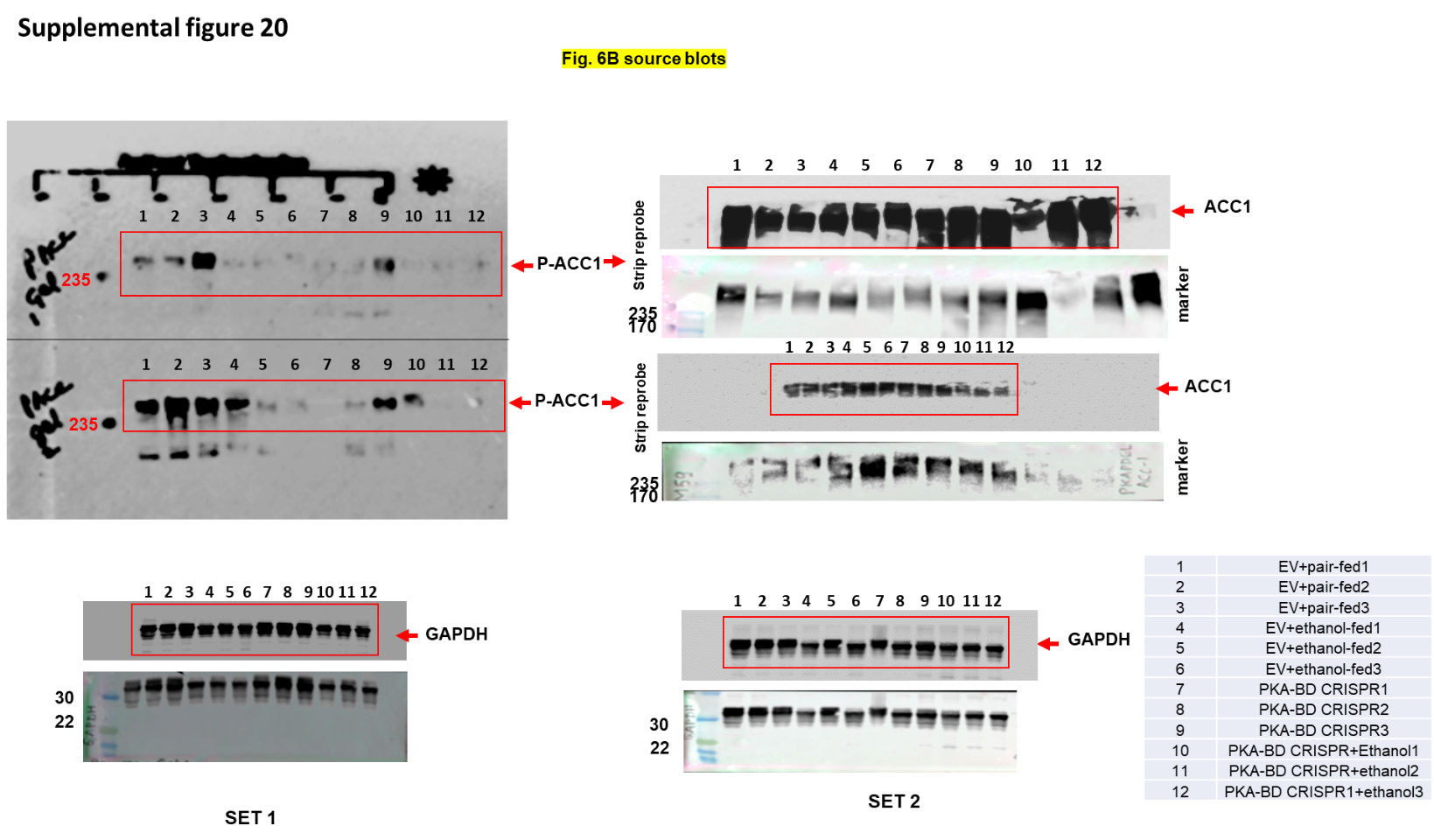


**Supplementary figure 20**. Uncropped blots for figure 6B with markers.

**Supplementary figure 21**. Uncropped blots for figure 7C and 7E with markers.

| **Reagent type** | **Designation** | **Sequence (5’-3’)/commercial source information** |
| --- | --- | --- |
| Edit-R Modified Synthetic crRNA, desalted/deprotected | Human *AKAP12* PKA-BD region crRNA | CAAAGTGAGTGTAGCAATTG |
| Custom ssDNA donor | Human *AKAP12* PKA-BD region CRISPR donor | TTGCCAGGAGGTCAAAGTGAGTGTAGCAATCGAGGATTTAGAGCCTGAAAATGGGTCCAAAACATCATCCAGACAGCCGTTGACC |
| Edit-R tracrRNA | Edit-R CRISPR-Cas9 Synthetic tracrRNA | Horizon discovery |
| SGRNA scaffold 1 | Mouse sgRNA 1 | CAAGATCTCAGGTTCAGCCC |
| SGRNA scaffold 1 | Mouse sgRNA 2 | TGCAAACTGGTCGACGGCTGT |
| Custom DNA donor | Mouse *Akap12* PKA-BD region CRISPR donor | AGGGTGAGGCTGGCCAGTTTGATGGAGAAAAAGTCAAAGACGGACAGTGTGTTAAAGAACTGGAGGTGCCTGTGCACACTGGACCCAACAGTCAAAAGACTGCTGACTTGACACGTGACAGTGAAGTAATGGAAGTGGCCAGATGTCAGGAAACTGAGAGTAATGAAGAACAGAGTATTAGCCCGGAGAAAAGAGAGATGGGAACCGACGTTGAAAAGGAGGAAACAGAGACCAAGACAGAGCAAGCCAGTGAAGAACATGAGCAGGAAACAGCTGCTCCTGAGCATGAAGGAACCCACCCTAAGCCAGTCCTGACAGCTGACATGCCTCACTCAGAGAGGGGAAAGGCACTGGGCAGCCTTGAAGGAAGCCCTTCTCTCCCAGACCAAGACAAAGCAGATTGCATAGAGGTTCAAGTTCAAAGCTCAGACACACCAGTCACTCAAACAACCGAAGCTGTGAAAAAGGTCGAAGAAACTGTGGCAACTTCAGAGATGGATGAAAGTTTGGAGTGTGCAGGTGCGCAATCATTACCAGCTGAGAAGCTCTCCGAAACCGGTGGCTACGGGACTCTTCAGCATGGAGAGGACACCGTGCCCCAGGGGCCTGAGTCTCAGGCAGAGTCCATCCCAATAATAGTAACTCCTGCTCCTGAAAGCATCCTACATTCTGACCTTCAAAGAGAAGTGAGCGCATCCCAGAAACAGAGATCAGATGAAGATAACAAGCCAGATGCTGGTCCTGATGCTGCCGGCAAGGAGAGTGCAGCAAGAGAGAAAATATTGAGGGCTGAACCTGAG |
| Amplicon sequencing primer | Human *AKAP12* PKA-BD forward primer | GAAGATGCTGTGCCCACA |
| Amplicon sequencing primer | Human *AKAP12* PKA-BD reverse primer | TCTGACTCCTCTTTGGC |
| Amplicon sequencing primer | Mouse *Akap12* PKA-BD forward primer | GCTCCTGAAAGCATCCTACAT |
| Amplicon sequencing primer | Mouse *Akap12* PKA-BD reverse primer | GGCACTGAGTGTTCCATCTT |

**Supplementary table 1:** Sequences and commercial sources of sgRNAs, donors and primers used in this paper.

| **Name** | **Supplier** | **Cat no.** | **Clone** |
| --- | --- | --- | --- |
| Anti-AKAP12 antibody (For PLA staining) | Abcam | ab49849 | mouse monoclonal, JP74 |
| Protein Kinase A regulatory subunit I alpha Antibody | Novus Biologicals | NBP1-47935 | ouse monoclonal, OTI6C7 |
| AKAP12 Polyclonal antibody | Proteintech | 25199-1-AP | rabbit polyclonal IgG |
| PRKAR1A Polyclonal antibody | Proteintech | 20358-1-AP | rabbit polyclonal IgG |
| PRKAR2A Polyclonal antibody | Proteintech | 10142-2-AP | rabbit polyclonal IgG |
| GAPDH Polyclonal antibody | Proteintech | 10494-1-AP | rabbit polyclonal IgG |
| Phospho-ACC1 (Ser79) Polyclonal antibody | Proteintech | 29119-1-AP | rabbit polyclonal IgG |
| ACC1 Polyclonal antibody | Proteintech | 21923-1-AP | rabbit polyclonal IgG |
| ADRP/Perilipin-2 Polyclonal antibody (PLIN2) | Proteintech | 15294-1-AP | rabbit polyclonal IgG |
| Recombinant Anti-PRKAR1A (phospho S77) antibody | Abcam | ab139682 | Rabbit monoclonal, EPMAYR1-127 |
| STK25 antibody | Proteintech | 25821-1-AP | rabbit polyclonal IgG |
| Albumin | Proteintech | 16475-1-AP | rabbit polyclonal IgG |
| Desmin | Proteintech | 16520-1-AP | rabbit polyclonal IgG |
| CD32b Antibody | Novus Biologicals | NBP2-14905 | rabbit polyclonal IgG |
| Clean-Blot™ IP Detection Reagent (HRP) | Life Technologies Corporation | 21230 |  |
| Goat Anti-rabbit IgG, HRP-linked Antibody | Cell signaling technology | 7074S |  |
| Goat anti rabbit IgG (Alexa Fluor® 488) | Abcam | ab150077 |  |
| Goat Anti-Mouse IgG H&L (Alexa Fluor® 488) | Abcam | ab150113 |  |
| Goat Anti-Rabbit IgG H&L (Alexa Fluor® 647) | Abcam | ab150079 |  |
| Goat Anti-Mouse IgG H&L (Alexa Fluor® 647) | Abcam | ab150115 |  |

**Supplemental Table 2**: Antibodies used in this study.

Data S1. (separate file)

**Differentially expressed protein (DEP) analysis.**

Out of a total of 1345 interacting partners identified, 352 exhibited a relative quantitation Fold-change (FC)of greater than 1.5 or less than 0.67 in ethanol group compared to control. 161 proteins exhibited a FC (ethanol/control) of greater than or equal to 1.5. 161 proteins exhibited a FC (ethanol/control) of less than or equal to 0.67. Normalized intensity= The total intensity normalized to the level of each protein.

Data S2. (separate file)

Differentially expressed genes sequencing analysis of mouse hepatocytes (DEGseq). The RNA sequencing data set of mouse hepatocytes consisted of 666 that were RNAs were upregulated by alcohol and 479 RNAs that were downregulated by alcohol. A subset of 586 genes were significantly modulated by AKAP12 overexpression in the presence of ethanol as depicted in the heatmap of figure 8a. The gene set (with the corresponding heatmap serial #) is presented under the ‘significant pathways’ red tab in the data S2 file. LOG2 (fold change) or LOG2 (FC) was calculated from the comparison of the ETHANOL+EV, AKAP12 OE and AKAP12 OE+ETHANOL groups to EV (CON) group, p<0.05.

Data S3. (separate file)

Differentially expressed genes sequencing analysis of mouse liver (DEGseq).

Out of 7733 RNAs exhibiting a significant change by alcohol in the RNA sequencing data set of mouse liver, 6104 RNAs were downregulated by alcohol and modulated by AKAP12 under basal or alcohol-stimulated conditions. A subset of 228 genes were significantly modulated by AKAP12 overexpression in the presence of ethanol as depicted in the heatmap of figure 8c. The gene set (with the corresponding heatmap serial #) is presented under the ‘significant pathways AKAP12 OE’ red tab in the data S3 file.

A subset of 91 genes were significantly modulated by AKAP12 PKA BD CRISPR in the presence of ethanol as depicted in the heatmap of figure 8d. The gene set (with the corresponding heatmap serial #) is presented under the ‘significant pathways PKA BD CR’ red tab in the data S3 file. LOG2 (fold change) or LOG2 (FC) was calculated from the comparison of the ETHANOL+EV, PKA BD CR and PKA BD CR+ETHANOL groups to EV (CON) group, p<0.05.

**Data S4**

**Quantitative raw data for graphs and anova/post-hoc statistical analysis for all figures**

Raw data for histograms and other graphs along with two- or three-way anova with Tukey’s post-hoc analysis is presented in data S4.
